# Ethanol extract of mulberry leaves partially restores the composition of intestinal microbiota and strengthens liver glycogen fragility in type 2 diabetic rats

**DOI:** 10.1186/s12906-021-03342-x

**Published:** 2021-06-14

**Authors:** Zhan-Zhong Liu, Qing-Hua Liu, Zhao Liu, Jia-Wei Tang, Eng-Guan Chua, Fen Li, Xue-Song Xiong, Meng-Meng Wang, Peng-Bo Wen, Xin-Yi Shi, Xiang-Yu Xi, Xiao Zhang, Liang Wang

**Affiliations:** 1 Xuzhou Infectious Diseases Hospital, Xuzhou, 221000 Jiangsu China; 2 Jiangsu Key Laboratory of New Drug Research and Clinical Pharmacy, Xuzhou Medical University, Xuzhou, 221000 Jiangsu China; 3 Department of Pharmaceutical Analysis, School of Pharmacy, Xuzhou Medical University, Xuzhou, 221000 Jiangsu China; 4 State Key Laboratory of Quality Research in Chinese Medicines, Macau University of Science and Technology, Avenida Wai Long, Taipa, Macau, 999078 China; 5 Faculty of Chinese Medicine, Macau University of Science and Technology, Avenida Wai Long, Taipa, Macau, 999078 China; 6 Department of Thyroid and Breast Surgery, Affiliated Hospital of Xuzhou Medical University, Xuzhou, 221000 Jiangsu China; 7 Department of Bioinformatics, School of Medical Informatics and Engineering, Xuzhou Medical University, Xuzhou, 221000 Jiangsu China; 8 Marshall Center for Infectious Diseases and Training, University of Western Australia, Perth, WA 6009 Australia; 9 Department of Laboratory Medicine, Huaiyin Hospital, Huai’an, 223300 Jiangsu China; 10 School of Life Science, Xuzhou Medical University, Xuzhou, 221000 Jiangsu China; 11 Institut Pasteur of Shanghai, Chinese Academy of Sciences, Shanghai, 200031 China

**Keywords:** Type 2 diabetes mellitus, Ethanol extract of mulberry leaves, Intestinal microbiota, 16 s rDNA, HFD/STZ treatment

## Abstract

**Background:**

Mulberry leaf as a traditional Chinese medicine is able to treat obesity, diabetes, and dyslipidemia. It is well known that diabetes leads to intestinal microbiota dysbiosis. It is also recently discovered that liver glycogen structure is impaired in diabetic animals. Since mulberry leaves are able to improve the diabetic conditions through reducing blood glucose level, it would be interesting to investigate whether they have any positive effects on intestinal microbiota and liver glycogen structure.

**Methods:**

In this study, we first determined the bioactive components of ethanol extract of mulberry leaves via high-performance liquid chromatography (HPLC) and liquid chromatography/mass spectrometry (LC/MS). Murine animal models were divided into three groups, normal Sprague-Dawley (SD) rats, high-fat diet (HFD) and streptozotocin (STZ) induced type 2 diabetic rats, and HFD/STZ-induced rats administered with ethanol extract of mulberry leaves (200 mg/kg/day). Composition of intestinal microbiota was analyzed via metagenomics by sequencing the V3-V4 region of 16S rDNAs. Liver glycogen structure was characterized through size exclusion chromatography (SEC). Both Student’s *t*-test and Tukey’s test were used for statistical analysis.

**Results:**

A group of type 2 diabetic rat models were successfully established. Intestinal microbiota analysis showed that ethanol extract of mulberry leaves could partially change intestinal microbiota back to normal conditions. In addition, liver glycogen was restored from fragile state to stable state through administration of ethanol extract of mulberry leaves.

**Conclusions:**

This study confirms that the ethanol extract of mulberry leaves (MLE) ameliorates intestinal microbiota dysbiosis and strengthens liver glycogen fragility in diabetic rats. These finding can be helpful in discovering the novel therapeutic targets with the help of further investigations.

**Supplementary Information:**

The online version contains supplementary material available at 10.1186/s12906-021-03342-x.

## Background

The prevalence of diabetes is expanding throughout the world due to the growing number of aged population and increasing urbanization, which imposes substantially negative impacts on the health of individual human beings and generates significantly economic burden on public healthcare systems. According to recent global estimates, diabetes prevalence in 2019 is around 9.3% (463 million people) and will increase to 10.2% (578 million) by 2030 and 10.9% (700 million) by 2045 [1]. 90% of cases are type 2 diabetes mellitus (T2DM), a progressive condition in which the body either becomes resistant to insulin and/or gradually lose the capacity to produce enough insulin [1]. Studies have already shown that, except for the typical feature of blood glucose imbalance, T2DM could also lead to other serious long-term complications, such as macrovascular disease, microvascular disease and neuropathy, etc. [2]. Currently, for the T2DM treatment, 50% of patients require the combinations of two or more types of non-insulin drugs while a third of patients will require insulin for lowering glucose [3]. Novel formulations and delivery methods for treating T2DM are also undergoing development [3].

Traditional Chinese medicine (TCM) is a well-developed and coherent system that has been existing in Chinese society for thousands of years [4]. Treatment of diabetes with TCM also has a long history [5]. A recent meta-analysis of randomized controlled trials of metabolic syndromes finds out that TCM is beneficial in regulating glucose and lipid metabolisms [6]. Studies also show that TCMs such as Xiaoke pill and Dahuang Huanglian Xiexin decoction (DHXD) are able to decrease blood glucose level and/or reduce body weight, together with improving other T2DM symptoms [5, 7]. Mulberry leaves (*Morus alba* L., abbr. MLs) are one of the most popular TCMs and are commonly used in the treatment of obesity and diabetes mellitus due to the abundant bioactive compounds such as polyphenols, alkaloids, flavonoids and polysaccharides, etc. [8]. Different forms of MLs such as powder [9], ethanol extract [10], and aqueous extract [11] have been studied, all of which suggest positive effects on the improvement of physiological conditions. In specificity, Suthamwong et al. used dried mulberry leaves (5% w/w) to feed db/db mice up to 20 weeks, which showed that both insulin levels and pancreatic β-cell mass were well maintained probably through the suppression of endoplasmic reticulum stress [12]. Sheng et al. found that mulberry leaf powder could alleviate the pathological conditions of streptozotocin-induced diabetic rats and modulate intestinal microflora [9]. In addition, Hu et al. discovered that the compound 1-Deoxynojirimycin isolated from mulberry leaves could significantly decrease serum glucose and insulin levels while improving serum lipid levels and reversing insulin resistance in streptozotocin-induced diabetic mice [13]. The effects of mulberry leaf on blood glucose response were also tested in healthy and diabetic human subjects, which confirmed that blood glucose levels were markedly reduced by mulberry leaf ingestion [14].

Liver glycogen plays important roles in blood glucose homeostasis. During T2DM progression, both liver glycogen accumulation and metabolism are abnormally changed [15]. Recently, a series of studies found that glycogen structure in T2DM liver was impaired and fragile, that is, larger glycogen α particles were easily dissociated into smaller β particles that were further degraded quickly, which might be a pathophysiological marker for T2DM at molecular level [16]. In contrast, glycogen structure in healthy liver had a diurnal alteration, that is, comparatively fragile during synthesis stage (high-glucose level after feeding) and stable during degradation stage (low glucose level after fasting) [16]. Thus, it was indicated that the consistent fragility of diabetic liver glycogen might be associated with the abnormally high blood glucose level [15, 17]. Since mulberry leaves had positive effects on reducing blood glucose level, it was hypothesized that administration of mulberry leaves might be able to repair liver glycogen fragility.

As for intestinal microbiota, its dysbiosis has been linked to the increasing prevalence of metabolic disorders including obesity, insulin resistance and type 2 diabetes, vice versa [18]. For example, it was shown that butyrate-producing bacteria were lower in T2DM subjects, so does the beneficial bacterium *Akkermansia muciniphila* [19]. In contrast, detrimental microbes such as *Fusobacterium nucleatum* and *Ruminococcus gnavus* were positively associated with T2DM, leading to increment of inflammatory cytokines [20]. Although previous studies confirmed that mulberry leaves could repair intestinal microbiota dysbiosis in diabetes, only dry powder or aqueous extract of mulberry leaves was used [9]. No effects of ethanol extract of mulberry leaves (MLE) on intestinal microbiota were reported, which is worthy of further investigation.

Taken together, we constructed a group of high-fat diet (HFD) and streptozotocin (STZ) induced type 2 diabetic Sprague-Dawley (SD) rats, which were then orally gavaged with MLE for 10 weeks. Intestinal microbiota and liver glycogen structure were examined in the normal control group, HFD/STZ-induced T2DM group, and MLE-fed HFD/STZ-induced T2DM group, respectively. Through this study, we confirmed the beneficial influences of MLE on T2DM treatment by partially normalizing intestinal microbiota composition. In addition, it was also found that MLE could repair glycogen structural fragility in diabetic liver, which indicated that there might be novel regulation pathways and potential drug targets for diabetic treatment.

## Methods

### Ethanol extraction of mulberry leaves

Mulberry leaves (2 kg) were purchased from Jikang Biotech Co. Ltd. in Anhui, China. The plant material was identified by Prof. Daoquan Tang from the School of Pharmacy at Xuzhou Medical University and the voucher specimen of the plant material was deposited in the publicly available herbarium at Xuzhou Medical University. During the first-time extraction, all clean-dried mulberry leaves were thoroughly mixed with 10 kg of 70% ethanol for a 90-min reflux extraction and produced 4.8 kg of extraction liquid. The mulberry leaves were then mixed with 10 kg of 70% ethanol for 90 min during the second-time reflux extraction and produced 6.1 kg of extraction liquid. The two portions of liquids were combined together and concentrated at 60 °C under vacuum condition (− 0.05Mpa) to generate 2 kg of concentrated liquid. Finally, a total of 135 g mulberry leaves powder was produced through the spray-drying technique.

### Component quantification of mulberry leaves

Polysaccharides content.

Accurately weigh 10 mg of glucose and dissolve it in 10 ml of pure water to prepare a glucose standard solution with the final concentration of 1 mg/mL. Take 0, 0.2, 0.4, 0.8, 1, 3, 5 mL of the standard solution in a 10 mL volumetric flask, add water to the mark, shake well to reach the final concentration of 0 mg/mL, 0.02 mg/mL, 0.04 mg/mL, 0.08 mg/mL, 0.1 mg/mL, 0.3 mg/mL, and 0.5 mg/mL standard solution. Take 1 mL of the standard solution in a test tube, add 1 mL of 5% phenol solution, shake well; add 5 mL of concentrated sulfuric acid, shake immediately, heat in a boiling water bath for 10 min, and cool to room temperature with ice water. Measure the absorbance value at 485 nm. Use the absorbance value as X-axis and the sample concentration as Y-axis to draw its standard curve. Weigh 0.5 g of MLE, add 0.5 mL of pure water to fully dissolve it, and then add an equal volume of 10% trichloroacetic acid solution to precipitate overnight. Centrifuge for 10 min (12,000 rpm, 4 °C), keep the supernatant, add an equal volume of 95% ethanol solution for precipitation overnight, and centrifuge for 10 min (12,000 rpm, 4 °C). Dissolve the precipitate in 1 mL of ultrapure water. The absorbance value is measured, and the polysaccharide content is calculated according to the standard curve.

#### Alkaloids content

100 mg of ethanol extract powder of mulberry leaves was dissolved in 20 mL of methanol for 24 h and then filtered; the filtrate was taken as the extract. The same procedure was conducted for two more times. Extracts were combined and concentrated, and then washed with 2% HCl solution to dissolve for 3 times. The combined acid solution was centrifuged at 5000 r/min for 10 min, which was adjusted to pH 10 with 10% NaOH, and then extracted for 3 times with equal volume of chloroform. The extracts were combined and concentrated to 10 mL. Standard matrine solution (Yifang Sicence and Technology, Tianjing, China) was prepared as references in the volumes of 0, 0.1, 0.2, 0.3, 0.4, 0.5 and 0.6 mL, which was filled up to 1 mL with chloroform, respectively. 5 mL of 2 × 10^− 4^ mol/L Bromothymol Blue (Sinoreagent, China) at pH 7 as color indicator and 5 mL of chloroform were then added and vortexed for 2 min. The mixed solution was put into separatory funnel, standing for 2 h. 4 mL of the chloroform layer was obtained, which was then added with 0.2 g of anhydrous sodium sulfate and well shaken. Leave it for 10 min, use the test sample without matrine as a blank control, and measure the absorbance at the maximum absorption wavelength. Linear regression equation was finally obtained. Pipette 1 mL sample test solution into a 20 mL test tube. Follow the above procedure to determine the absorbance. According to linear regression equation, alkaloids amount in the sample was identified.

#### Polyphenols content

Weigh 100 mg of ethanol extract of mulberry leaves into a centrifuge tube, and then add 5 mL of pre-heated 70% methanol aqueous solution (70 °C). Stir evenly with a glass rod and immediately transfer the solution to 70 °C water bath for 10 min extraction (stir every 5 min). After cooling down to room temperature, centrifuge the solution at 4000 r/min for 10 min, and transfer the supernatant to a 10 mL volumetric flask. Residual sample was re-extracted with 5 mL of 70% methanol aqueous solution. The extracts were combined to a constant volume of 10 mL, well shaken, and then passed through a 0.45 μm filter membrane for testing. Pipette 1, 2, 3, 4, and 5 mL 1000 mg/L gallic acid standard stock solution to 100 mL volumetric flasks, respectively, which were then diluted to the concentration of 10, 20, 30, 40, and 50 μg/mL. Pipette to transfer 1 mL of each of the gallic acid working solution, water, and test solution into the Graduated test tubes. In each test tube, add 5 mL of 10% folin and shake well. After 3 to 8 min of reaction, add 4 mL of 75% sodium carbonate solution and water to make up to scale and shake well. Place the solution at room temperature for 60 min, use a 10 mm cuvette to measure the absorbance with a spectrophotometer at a wavelength of 760 nm. According to the absorbance of the gallic acid working solution and the concentration of each working solution, a standard curve was drawn. Polyphenol content was determined by colorimetry. The following formula was used for the calculation:

$$ X=\frac{\left(A-{A}_0\right)\times V\times d\times 100}{k\times m\times {10}^6} $$

X: content of total polyphenols in the test sample (g/100 g); A: absorbance of the test sample solution; A_0_: absorbance of blank sample; k: slope of the standard curve of gallic acid; m: test sample weight (g); V: test sample volume (mL); d: dilution factor.

#### Flavonoids content

Precisely weigh 10 mg of the rutin as a reference substance. Add 10 mL of absolute ethanol and dissolve the rutin ultrasonically with a final concentration of 1 mg/mL. Take the rutin reference solution 0, 0.2, 0.5, 1, 2, 3, and 4 mL to a 10 mL volumetric flask, respectively. Add water to the mark and shake well to reach the final concentration of 0, 0.02, 0.05, 0.1, 0.2, 0.3 and 0.4 mg/mL. Add 4 mL of 5% sodium nitrite solution and let stand for 6 min, then add 0.4 ml of 10% aluminium nitrate solution to shake well. Let the solution stand for 15 min. Measure the absorbance value at 510 nm and draw the standard curve with the absorbance value as X-axis and the sample concentration as Y-axis. Weigh 200 mg of MLE powder, dissolve it with 1 ml of absolute ethanol ultrasonically for 10 min, and then centrifuge for 10 min (12,000 rpm, 4 °C). Keep the supernatant and measure the absorbance. The total flavonoid content in the sample is calculated according to the standard curve.

#### Crude protein content

Crude protein was calculated as N × 6.25 by Kjeldahl Methods [21]. For details, please refer to the Official Methods of Analysis by AOAC International Press (Washington DC, 2005) [21].

#### Liquid chromatography/mass spectrometry (LC/MS) analysis of all components

Functional components of the MLE were detected by high-performance liquid chromatography (HPLC) with an Agilent 1100 HPLC system (Agilent Technologies Inc., California, USA), together with Waters Xevo G2XS QT of mass spectrometry system (Waters Corporation, Milford, MA, USA). Liquid phase conditions: mobile phase: phase A: water, phase B: acetonitrile. Gradient: 0–1 min: A phase 95%, B phase 5%; 1–8 min: A phase 0%, B phase 100%; 8–11 min: A phase 0%, B phase 100%; 11–12 min: 95% for phase A and 5% for phase B. Flow rate: 0.3 mL/min. Injection volume: 2 μL. Column: BEH C18 1.7 μm 2.1 × 50 mm Column. Mass spectrometry conditions: capillary voltage: 3.0 KV; sample cone: 40 V; Source temperature: 100 °C; desolation temperature: 400 °C; cone gas (cone gas: 50 L/h; desolation gas: 800 L/h. For a complete list of all the components detected by LC/MS technique in the ethanol extract of mulberry leaves, please refer to Supplementary Table 1.

### Animal model

Three groups of SD rats were used in this study: normal control (NC), type 2 diabetes mellitus (T2DM), and T2DM group treated with MLE. The T2DM model rats were constructed by following the well-established HFD/STZ method [15]. 18 male SD rats with body weight around 200 g were purchased from the Center for Animal Experiment of Xuzhou Medical University. The rats were bred in a standard specific pathogen free (SPF) animal room with 2 rats per cage. The temperature was controlled at 22 ± 1 °C with a 12 h dark/light cycle (light on at 6 am) [15]. All rats had ad libitum access to water and standard pellet diet (Jiangsu Synergetic Biotechnology Co., Ltd.) during their adaption to the lab environment for 1 week (average body weight≈270 g). The standard pellet consists of 10% water, 20% crude protein, 4% fat, and 66% carbohydrate. Rats were then randomly divided into NC group (*n* = 6), T2DM group (n = 6), and MLE group (n = 6) (200 mg/kg oral administration) as routinely required. T2DM and MLE groups were fed with 40% high fat diet (20% crude protein, 40% fat, 40% carbohydrate) that was purchased from Shanghai Proton Biotechnology Co., Ltd., and control groups were given a normal pellet diet for 5 weeks. At the 6th week, the experimental groups (T2DM and MLE) were fasted for 12 h, followed by a single intraperitoneal injection of freshly prepared STZ (35 mg/kg) dissolved in 0.1 M citrate buffer (pH 4.5), while the control group (NC) was given an equal amount of citrate buffer only. After the model construction, the MLE group was given ethanol extract of mulberry leaves via daily gavage at the dosage of 200 mg/kg [22].

#### Measurement of physiological indicators

Fasting blood glucose level was measured from tail veins with the aid of a glucometer (Jiangsu Yuwell Medical Equipment Co., Ltd., China) at the 7th week, that is, 7 days after STZ injection, and rats with blood glucose levels greater than 250 mg/dL were considered as successful T2DM models [15]. An oral glucose tolerance test (OGTT) was also performed at one-week after STZ injection to validate the successful induction of T2DM rats, for which blood glucose concentrations at 0, 5, 10, 20, 30, 60 and 120 min were measured. OGTT was also performed after 10-week MLE treatment [15]. Values are expressed as mean ± SEM (standard error of the mean). Determinations of plasma glucose (Nanjing Jiancheng Bioengineering Institute, China), plasma triglycerides (Nanjing Jiancheng Bioengineering Institute, China), plasma total cholesterol (Nanjing Jiancheng Bioengineering Institute, China) and high/low density lipoprotein (Shanghai Huaxuan Biotechnology Co. Ltd., China) were carried out 7 days before STZ injection, 7 days after STZ injection, and after 10-week MLE treatment for all three groups by following manufacturer’s instructions [15]. Feces of all rats were collected at the last week of the experiment and frozen in − 80 °C freezer for further analysis. All rats were euthanized through single intraperitoneal injection of sodium pentobarbitone (200 mg/ml) in a dosage of 200 mg/kg at week 17. Liver was then removed immediately and snap-frozen in liquid nitrogen for further analysis. After the experiments, animal bodies were taken care of by Animal Experiment Center of Xuzhou Medical University. Ethical approval for this study was obtained from the Laboratory Animal Welfare and Ethics Committee of Xuzhou Medica University, China. The animal care and experimental procedures were carried out in accordance with the Guidelines of the Animal Care and Use Committee of Xuzhou Medical University, China.

### Liver glycogen extraction

1.5 g of rat liver was first homogenized in 10 mL of glycogen extraction buffer as described in a previous study [15]. Samples were centrifuged at 6000 g for 10 min at 4 °C, the supernatants of which were then centrifuged at 360,000 g for 2 h at 4 °C. The pellets were resuspended in deionized water and layered over an 8 mL stepwise sucrose gradient (37.5 and 75% in ddH_2_O). Samples were then centrifuged at 360,000 g for 2.5 h at 4 °C. The pellets were resuspended in 200 μL of ddH_2_O. 800 μL of absolute ethanol was added to precipitate glycogen. The samples were centrifuged at 4000 g for 10 min and the pellets dissolved in 200 μL of ddH_2_O and vacuum freeze-dried.

### Liver glycogen quantification

The glycogen content of each liver sample was determined as previously described [15]. Briefly, amyloglucosidase (3260 U/mL) from *Aspergillus niger* was first used to degrade glycogen into glucose, which was then measured by the glucose oxidase/peroxidase assay kit (GOPOD, Megazyme). Glycogen content was calculated based on a standard curve constructed by reacting D-glucose of various concentrations with GOPOD reagent. All samples and reference substances were measured in triplicate. However, for NC and MLE group, only five rats have sufficient glycogen extracted from liver samples for further structural analysis.

### Glycogen structure characterization

Glycogen was analysed by using aqueous size exclusion chromatography (SEC) to obtain molecular size distributions. Sodium nitrate solution (50 mM) containing 0.02% sodium azide (w/w) was used as the mobile phase. 2 mg/mL glycogen was dissolved in 50 mM ammonium nitrate with 0.02% sodium azide and DMSO in a thermomixer for 8 h at 80 °C, respectively. Glycogen was then precipitated with absolute ethanol. The resulting suspension was centrifuged at 4000 g and the pellet was washed with ethanol for two times before water dissolution and lyophilization. Samples were injected into an Agilent 1260 Infinity SEC system (Agilent, Santa Clara, CA, USA) by following the procedures as previously described [23].

### Characterization of intestinal microbiota composition

16S rDNA gene amplicon (V3-V4 region) was sequenced on Illumina MiSeq platform for determining the intestinal microbiota composition. The raw reads files and sequence files with assembled reads after quality control and filtering were available under request. Faecal samples were collected from three groups (NC, T2DM, MLE) after 10-week treatment of the ethanol extract of mulberry leaves on T2DM rat models, which were then stored immediately at − 80 °C freezer. DNA was extracted by using Qiagen DNA Stool Mini Kit and quantified by NanoDrop ND-1000 spectrophotometer, V3-V4 region of which was amplified and was then sequenced by Illumina MiSeq at Sangon Biotech (Shanghai) Co., Ltd. Six samples from each group were used for the intestinal microbiota analysis via Mothur http://www.mothur.org [24]. Alpha diversity is conducted for analysing the complexity of species diversity for each group through the calculation of community richness (Chao1 estimator) and community diversity (Shannon index). Beta diversity (Unweighted UniFrac Emperor PCoA) was used to evaluate the differences in the species complexity. Cluster analysis was preceded by principal component analysis (PCoA). Community structure variance analyses were also performed at phylum and genus level.

### Statistical analysis

Excel and R packages were used for statistical analysis. Tabulated data were presented in the form of the mean value and standard deviation (STD). Two-tailed unequal variance Student’s *t*-test was used for pair-wise comparison (*, *P*-value < 0.05; **, *P*-value < 0.01; ***, *P*-value < 0.001). Tukey’s Honestly Significant Difference (HSD) test was performed for multi-group analysis, which compared the means of every group to the means of every other group simultaneously. Means denoted by a different letter indicated significant differences between groups (*P*-value < 0.05).

## Results

### Composition of mulberry leaves

Mulberry leaves contain many chemical constituents, which possesses various beneficial effects, such as anti-hyperglycaemic, anti-hyperlipidaemic and anti-obesity effects, etc., according to previous studies [25]. In addition, mulberry leaves are one of the most commonly used TCM for the treatment of diabetes mellitus and its associated complications. In particular, major bioactive components in mulberry leaves, such as alkaloids, flavonoids, polyphenols and polysaccharides have proved effects on the regulation of blood glucose levels [8]. In this study, we used a variety of standard methods to quantify the contents of the above-mentioned four groups of bioactive components, together with crude protein content, which was summarized in Table 1. According to the results, the concentration of polysaccharides was 19.2 mg/g mulberry leaves powder. The level of polyphenols reached 45 mg/g mulberry leaves powder. In addition, mulberry leaves were rich in flavonoids (78.4 mg/g) and alkaloids (22.7 mg/g). As for crude proteins, its concentration was 198 mg/g mulberry leaves powder.

**Table 1:** Chemical composition of major bioactive components in ethanol extract of mulberry leaves

Substance	Concentrations
Polysaccharides (mg/g) (Dextrose equivalent)	19.2
Proteins (mg/g)	198
Polyphenols (mg/g) (Gallic acid equivalent)	45
Flavonoids (mg/g) (Rutin equivalent)	78.4
Alkaloids (mg/g) (Matrine equivalent)	22.7

### HFD/STZ-induced type 2 diabetic rats

**Fig. 1:**
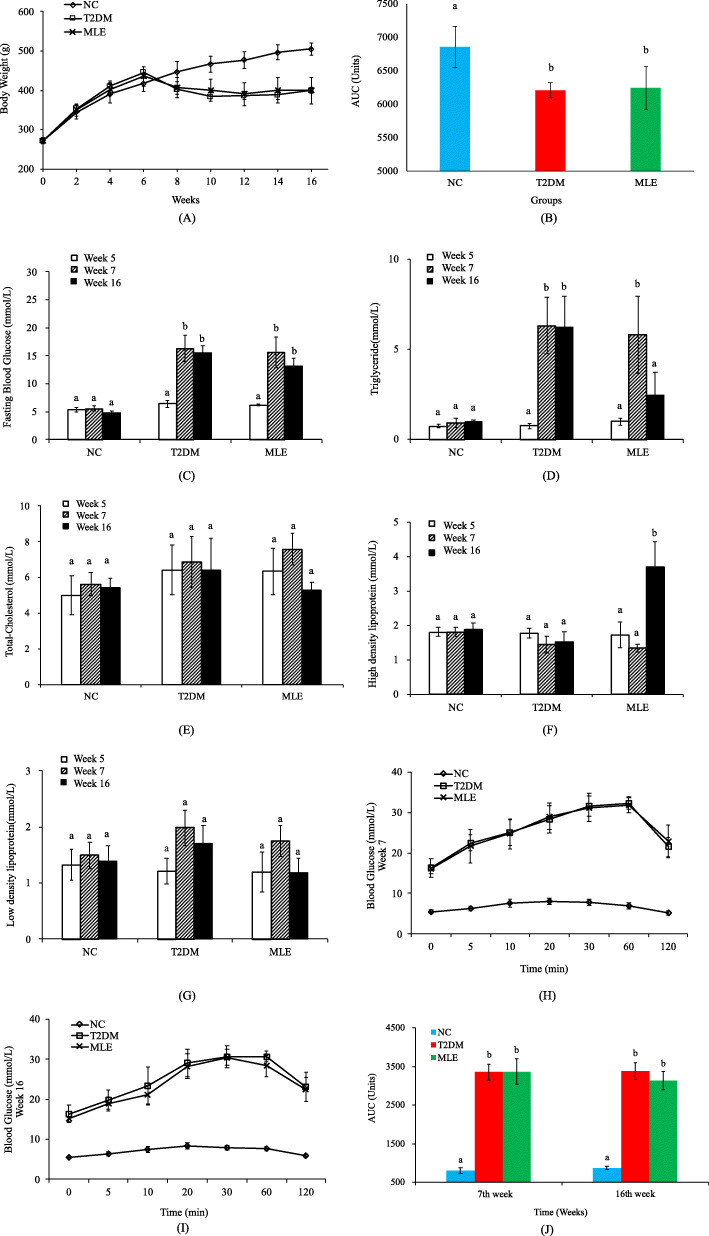
Comparison of indices during the construction of the HFD/STZ-induced T2DM rat models and the treatment effects of MLE administration at week 5 (before STZ injection), week 7 (after STZ injection) and week 16 (after MLE treatment). (A) Body weight. (B) Areas under curves of body weights (C) Fasting blood glucose. (D) Triglyceride. (E) Total cholesterol. (F) High-density lipoprotein. (G) Low-density lipoprotein. (H) OGTT (week 7). (I) OGTT (week 16). (J) Areas under curves of OGGTs. For each group, six SD rats were used for all the calculations. Statistical analysis was conducted via Tukey’s test and means denoted by a different letter indicated significant differences between groups (*P*-value < 0.05)

Body weights (BWs) were monitored on a bi-weekly basis (Fig. 1A). For the first 6 weeks, average body weights of the three groups gradually increased while HFD-fed rats had comparatively higher body weights. After STZ injection, one of the HFD-fed groups was administered with ethanol extract of mulberry leaves, hence the MLE group. Meanwhile, body weights of both HFD and MLE groups dropped sharply. Difference of body weights between normal group and HFD-group/MLE-group is statistically significant (Fig. 1B), which is consistent with previous studies [15]. In order to confirm the successful construction of T2DM model, several indicators were assessed before and after STZ injection, which included fasting blood glucose (FBG) (Fig. 1C), triglyceride (TG) (Fig. 1D), total cholesterol (TC) (Fig. 1E), high-density lipoprotein (HDL) (Fig. 1F), and low-density lipoprotein (LDL) (Fig. 1G). In addition, oral glucose tolerance test (OGTT) was also measured (Fig. 1H-I) and the corresponding areas under curves were calculated (Fig. 1J). After STZ injection, FBG and TG significantly increased while the levels of TC (Fig. 1D) and LDL (Fig. 1G) also rose with no statistical significance. It was worthy of mentioning that, before STZ injection, HFD-feeding alone led to the increment of total cholesterol (Fig. 1E). As for HDL, its level in T2DM group was apparently reduced at week 7 (Fig. 1F). After STZ injection at week 7, OGTT test showed that the two HFD-groups had significant increment of areas under curve (AUC) when compared with that in the NC group. Combined these together, rats used in this study were successfully constructed as T2DM models after HFD feeding and STZ injection.

### Effects of mulberry leaves on physiology

After 10-week administration of mulberry leaves, physiological indicators were evaluated and compared among groups, which included BW, FBG, TG, TC, HDL, LDL and OGTT. T2DM and MLE had the same BWs on average, which was 399.7 g, while NC had a much higher averaged BW at 504.9 g. Thus, mulberry leaves did not have obvious impact on body weight when compared with T2DM, though BWs in MLE at week 10 (400.3 g vs. 383.6 g) and week 14 (400.3 vs. 387.9 g) were higher than those in T2DM (Fig. 1A). As for FBG, MLE had lower value than that in T2DM with no statistical significance, which suggested that mulberry leaves might have positive effects on blood glucose control (Fig. 1C). Both intra-group and inter-group comparisons for TG, TC, and LDL found similar patterns as that in FBG. In specificity, all of the indicators decreased at week 16 when compared with those at week 7 within the group of MLE. However, only TG showed significant reduction (Fig. 1D). As for the inter-group comparison, TG was significantly reduced between T2DM and MLE at week 16 while TC and LDL were lower in MLE than T2DM with no significance. As for HDL, it was significantly increased at week 16 when compared both within and between groups (Fig. 1F). OGTT was also measured at week 16. Decreased but not significantly different AUC was observed in MLE group when compared with that in T2DM group, which indicated that there might be a better response toward glucose administration after treatment of the ethanol extract of mulberry leaves. Taken together, administration of mulberry leaves had positive effects on T2DM and the physiological conditions of T2DM rats were improved.

### Intestinal microbiota diversity and composition

#### Operational taxonomic units

**Fig. 2:**
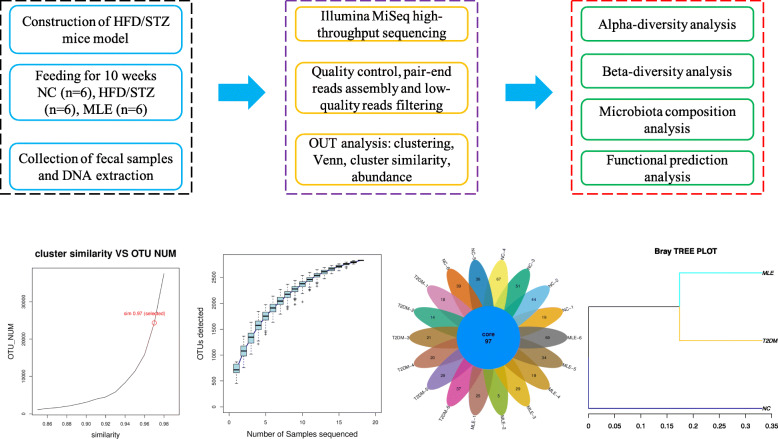
Illustration of 16 s rDNA gene amplicon sequencing and operational taxonomic units (OTUs) analysis. (A) Flow chart of 16 s rDNA gene amplicon sequencing. (B) The relationship between cluster similarity and OTU numbers. (C) Species accumulation curves. (D) Venn diagram for the distribution of core and unique OTUs in each sample. (E) Bray-Curtis tree for group distances based on OTU abundance

Procedures for microbiota sequencing, diversity analysis, and composition analysis were summarized in Fig. 2A. Operational taxonomic units are used to categorize bacteria based on sequence similarity. OTU cluster is defined by a 97% identity threshold of the 16S rDNA gene sequences to distinguish bacteria at the genus level while species separation requires a higher threshold (Fig. 2B). Species accumulation curves are used to describe the increase of species as the amount of sample increases. It is an effective tool for investigating the species composition and predicting the species abundance in the sample. In this study, the species accumulation curve tends to be flat with the increment of sample sizes, which indicates that the sample amount is sufficient (Fig. 2C). As for the Venn Diagram, it is used to count the number of shared and unique OTUs in the sample. According to the result, all the samples from the 3 groups share 97 OTUs while each sample has different number of unique OTUs ranging from 5 to 69 (Fig. 2D). In addition, sample clustering tree diagram can intuitively reflect the similarities and differences between multiple samples through the branch structure. By using the vegan package in R, we calculated the distance between the three groups according to the UPGMA (Unweighted pair group method with arithmetic mean) algorithm. The relationship of the three groups were visualized in Bray-Curtis tree form, according to which, groups of NC, T2DM, and MLE were well separated (Fig. 2E).

#### Microbiota diversity

**Fig. 3:**
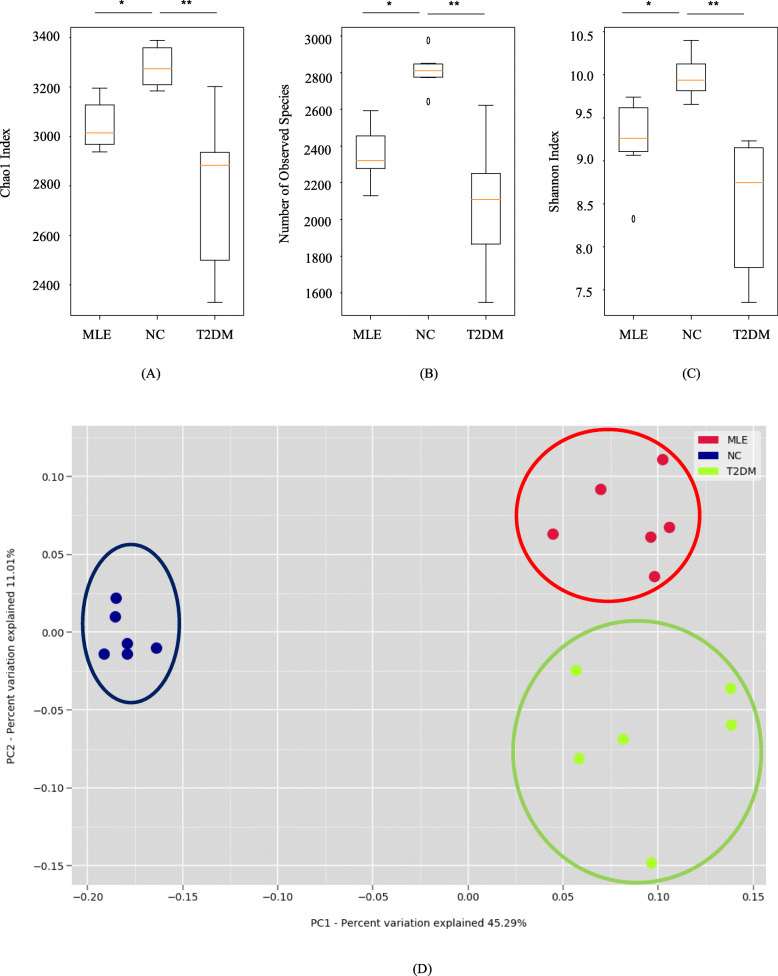
Alpha-diversity and beta-diversity analyses revealed significant differences among the microbiota of the three groups (NC, T2DM, and MLE). (A) Chao1 index. (B) Number of observed. (C) Shannon index. (D) Unweighted unifrac emperor PCoA. Statistical analysis was conducted via two-tailed unequal variance Student’s *t*-test (*, *P*-value< 0.05; **, *P*-value< 0.01; ***, *P*-value< 0.001)

In order to better understand the effects of MLE on intestinal microbiota, a series of analyses in terms of diversity and composition of intestinal microbiota were conducted and compared among NC, T2DM and MLE groups. For alpha-diversity analysis, Chao1 richness estimator is an index of community richness, which is normally used to estimate the number of operational taxonomic units (OTUs) contained in a sample. The larger the value, the more species contained in the sample. According to the results, it was shown that NC has the highest number of OTUs (*n* = 3282) on average, while OUTs in T2DM (*n* = 2770, *P* < 0.01) and MLE (*n* = 3046, *P* < 0.05) were significantly lower (Fig. 3A). Number of observed species indicates the number of species contained in the sample. The higher the value, the higher the species richness of the sample. In Fig. 3B, it was obvious to see that NC (*n* = 2810) had more species than MLE (*n* = 2354, *P* < 0.05) and T2DM (*n* = 2078, *P* < 0.01), though the number of species in MLE was closer to NC and higher than T2DM. As for Shannon index, it assesses the diversity and uniformity of species composition in the sample. The larger the value, the more abundant species in the environment, and the more even the distribution of species is. Based on the results, it was indicated that species composition was more abundant and more evenly distributed in NC. Meanwhile, T2DM showed reduced abundance and uneven distribution while the index in MLE was increased (Fig. 3C). Unweighted UniFrac PCoA clusters the samples based on the measures of the difference coefficients among samples. The closer the samples, the smaller the differences in species diversity are. According to Fig. 3D, individual samples in the three groups were well separated into three clusters, which indicated compositional differences of intestinal microbiota among the three groups. In sum, mulberry leaves had beneficial effects on intestinal microbiota, which improved the number, abundance, and distribution of bacterial species in intestinal microbiota.

#### Microbiota composition

**Fig. 4:**
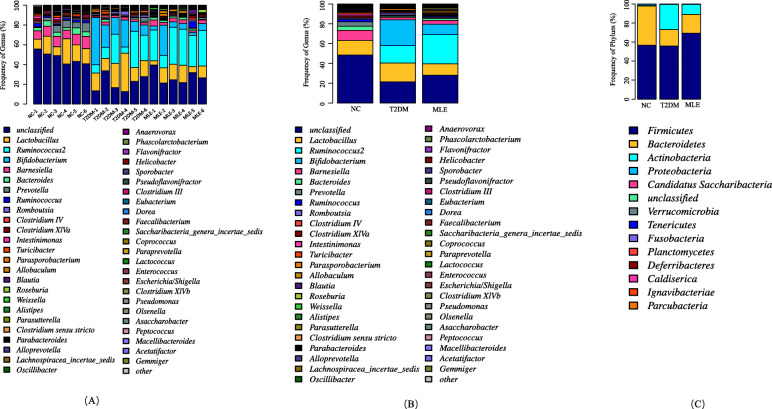
Bacterial compositions of the intestinal microbiota. (A) Composition of intestinal microbiota in the three groups at phylum level. (B) Composition of intestinal microbiota in the three groups at genus level. (C) Composition of intestinal microbiota in each sample at genus level

Through the cluster of OTUs and taxonomy annotation, clean reads were classified into 14 taxa at phylum level and 148 taxa at genus level. In this annotated dataset, *Firmicutes* (NC 55.72%, T2DM 58.41%, MLE 70.78%) and *Bacteroidetes* (NC 42.27%, T2DM 18.27%, MLE 19.31%) were the most abundant phyla of the OTUs in all groups, except for the phylum *Actinobacteria* in T2DM (22.57%), the abundance of which is higher than *Bacteroidetes* (18.27%). Thus, HFD/STZ treatment resulted in apparent divergences in the community structure of the intestinal microbiota between healthy group (NC) and diabetic group (T2DM). After 10-week administration of the ethanol extract of mulberry leaves, the abundance of *Bacteroidetes* (↑, 1.06-fold) was increased while that of *Actinobacteria* (↓, 41.91%) dropped when compared with T2DM group, which makes the microbiota composition of MLE group much closer to that in the intestinal microbiota of NC group than that in T2DM group. However, the level of *Firmicutes* was increased further to 70.78% in MLE group. Thus, ethanol extract of mulberry leaves could, at least partially, restore the disordered community structure of the intestinal microbiota in T2DM rats (Fig. 4A). For detailed composition of intestinal microbiota at phylum level, please refer to Supplementary Table 2. At genus level, T2DM group had remarkably elevated levels of *Bifidobacterium* (↑, 2240-fold), *Ruminococcus2* (↑, 280-fold), *Romboutsia* (↑, 1.53-fold) and *Lactobacillus* (↑, 1.31-fold) when compared with the NC group. Meanwhile, the levels of *Bacteroides* (↓, 27.09%), *Barnesiella* (↓, 24.78%) and *Ruminococcus* (↓, 18.78%) were reduced (Fig. 4B). After 10-week treatment of mulberry leaves, part of the intestinal microflora of HFD/STZ-treated mice was restored. At the genus level, the abundance of *Bifidobacterium* (↓, 41.43%), *Romboutsia* (↓, 37%) and *Lactobacillus* (↓, 57.05%) were greatly reduced in MLE group when compared with T2DM while the levels of *Bacteroides* (↑, 1.61-fold), *Barnesiella* (↑, 1.47-fold) and *Ruminococcus* (↑, 3.625-fold) were apparently improved. However, the percentage of *Ruminococcus2* was further improved, reaching to 30.86%. For detailed composition of intestinal microbiota at genus level, please refer to Fig. 4C and Supplementary Table 3. In sum, HFD/STZ-induced T2DM rats were altered in their intestinal microbiota composition, which could be partially repaired by administration of mulberry leaves. Taken together, these data showed that the treatment of mulberry leaves could partially reverse the dysbiosis of intestinal microbiota.

### Alteration of liver glycogen structure

**Fig. 5:**
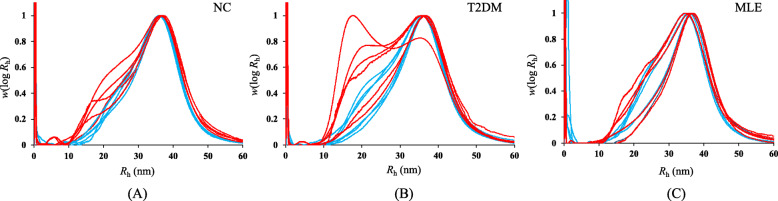
Size exclusion chromatography of glycogen particles before DMSO treatment (blue line) and after DMSO treatment (red line) in (A) NC group (*n* = 5), (B) T2DM group (*n* = 6), and (C) MLE group (n = 5). X-axis is the hydrodynamic radius (*R*_h_) of glycogen particles while Y-axis is the weight distributions, *w* (log *R*_h_), being the relative weight of molecules with *R*_h_, normalized to the maximum, as a function of the SEC separation parameter *R*_h_

The structure of liver glycogen consists of three hierarchical levels, that is, level 1 (γ-particle), level 2 (β-particle), and level 3 (α-particle). γ-Particles are normally 3 nm in size and are highly electro-dense; β-particles are 20–30 nm in diameter; while α-particles are assembled from β-particles with unknown mechanisms [26]. In mammalian liver, glycogen mainly consists of large rosette-shaped α-particles. Recently, the structure of glycogen α-particles was found to be fragile in diabetic liver, which was considered as a pathophysiological marker for diabetes [15]. A couple of studies also confirmed that diabetic drugs with a function of blood glucose control could be able to restore glycogen fragility. In this study, we analyzed the structure of purified liver glycogen particles from NC (*n* = 5), T2DM (*n* = 6) and MLE (n = 5) groups in terms of particle size distribution and structural stability (Fig. 5). According to the results, size distribution of glycogen particles from NC group showed a unimodal pattern with peak value of hydrodynamic radius (*R*_h_) at 36.93 nm (Fig. 5A, blue line). After DMSO treatment, the distribution pattern is similar to that in raw state and peak value of *R*_h_ did not change (Fig. 5A, red line). Thus, it indicates that the structure of liver glycogen in NC group is stable. As for T2DM, liver glycogen shows structural fragility due to distribution pattern change after DMSO treatment (Fig. 5B). In particular, typical bi-modal distribution pattern has been observed after DMSO treatment, which indicates the dissociation of fragile α-particles into smaller β particles. After mulberry leaf treatment, liver glycogen fragility returns to th structural stability state as shown in Fig. 5C. Thus, according to the SEC results, it is concluded that ethanol extract of mulberry leaves is able to strengthen structural fragility of diabetic liver glycogen particles and restore it to stable state.

## Discussion

Type 2 diabetic mellitus is a metabolic disorder that affects a very large number of people at all ages all over the world. If left untreated, T2DM patients can develop severe complications such as kidney damage, eye damage, and increased risks for heart disease or stroke due to the constant high blood glucose level [27]. In this study, we constructed a group of T2DM rat models through HFD feeding and STZ injection as previously described [15]. Hyperglycemia phenotype was successfully induced, which is consistent with previous studies [9]. Mulberry leaves have been widely used in China and other Asian nations as a traditional medicine for the treatment of glucose abnormalities and other diseases [28]. In addition, previous studies showed that mulberry leaves were used in a variety of forms, such as dry powder, water extract, ethanol extract and methanol extract, etc., in which the composition of bioactive components were vastly different and could lead to different functions [9, 11, 21, 29]. Moreover, nutritional components of mulberry leaves vary due to the varieties of growth conditions, which may also have impacts on its functions [21, 30]. Thus, it is necessary to quantify the functional components in mulberry leaves.

Animal and human studies indicate that MLE has potential benefits in T2DM [28]. In this study, we quantified several major bioactive components in the ethanol extract of mulberry leaves, such as polysaccharides (1.92%), polyphenols (4.5%), flavonoids (7.84%) and alkaloids (2.27%), which were all well known to have pharmacological effects on the treatment of T2DM conditions [31–33]. In specificity, polysaccharides extracted from mulberry leaves are able to decrease blood glucose level, improve glucose tolerance, and increase liver glycogen content in diabetic mice [34]. Recent studies also suggest that the potential molecular mechanism of hypoglycemic activities of polysaccharides from mulberry leaves involves inhibition of pancreatic islet cell apoptosis and amelioration of insulin secretory capacity [35]. As for polyphenols, abundant varieties and quantities in mulberry leaves have been confirmed, which have significantly beneficial effects of lowering blood glucose level in diabetic conditions [33]. It is found that polyphenols play a key role in reducing postprandial glucose levels by inhibiting mouse maltase activity and glucose transportation [36]. In addition, polyphenols can also delay aging, improve oxidative stress resistance, and reduce fatty acid storage in vivo [37]. Flavonoids have also been confirmed to effectively ameliorate glucose uptake and improve insulin resistance through AMPK-PGC-1α signalling pathway, which supports the therapeutic effects of flavonoids on T2DM [32]. In terms of total alkaloids in mulberry leaves, studies show that it has hypoglycemic effects in STZ-induced mice through inhibition of α-glucosidase activity [34]. In this study, we confirmed that ethanol extract of mulberry leaves could decrease the levels of blood glucose, TG, TC and LDL while improving the level of HDL. Thus, mulberry leaves possessed important functions in antihyperglycemic and antihyperlipidemic activities, which is consistent with previous studies.

Many studies have also investigated the composition of intestinal microbiota of diabetic animal models and how ingestion of different forms of mulberry leaves alters intestinal microbiota in these models [9]. Cumulative evidence has demonstrated that composition of the intestinal microbiota is different between healthy and diabetic individuals [9]. In this study, we showed that the compositions of intestinal microbiota in the three rat groups, NC, T2DM and MLE, were rather different. Both HFD/STZ and MLE treatment could greatly change the composition of intestinal microbiota. At phylum level, we observed that the reduction of *Bacteroidetes* from 42.27% in NC to 18.27% in T2DM. Such reduction was consistently associated with hyperglycemia [38]. After the treatment of mulberry leaves, abundance of *Bacteroidetes* was somewhat but not significantly improved to 19.31%. As for *Actinobacteria*, it jumped from 0.23% in NC group to 22.57% in T2DM group while mulberry leaves greatly reduced its percentage to 9.46%. According to previous studies, relative abundance of *Actinobacteria* in adult human beings was positively associated with diabetic patients [38]. As for *Firmicutes*, its percentage was only slightly increased from 55.72% in NC group to 58.41% in T2DM group but greatly increased to 70.78% in MLE group, though previous studies showed that the reduction of *Firmicutes* abundance was a key pathway for the antiobesity effects [10]. Although some studies used *Firmicutes/Bacteroidetes* ratio as a hallmark of obesity and diabetes, new results suggest that it is difficult to associate the *Firmicutes/Bacteroidetes* ratio with a determined health status [39]. At the genus level, our results showed that diabetic rats had a remarkably elevated level of *Bifidobacterium*, *Ruminococcus2*, *Romboutsia*, and *Lactobacillus* when compared with healthy rats. After 10-week mulberry leaf treatment, microbiota alteration caused by the influence of STZ treatment was significantly reversed. In particular, the level of *Bifidobacterium* dropped to 41.43%. Taken together, these data indicated that the treatment of ethanol extract of mulberry leaves could be able to partially reverse the changed intestinal microbiota from abnormal state in diabetic rats to normal state in healthy rats.

The consistent fragility of liver glycogen structure was initially discovered in T2DM mice [40]. It was later found that liver glycogen fragility was a pathophysiological signature for both T1DM and T2DM mice at molecular level [16, 41]. In addition, the structure of healthy liver glycogen experiences a diurnal change between stable and fragile states, that is, fragile during night-time feeding stages (glycogen synthesis) and stable during day-time fasting stages (glycogen degradation) [16]. Currently, the molecular mechanisms of liver glycogen fragility are not clear and are under intensive investigations [15]. Previously, active components such as astragalus polysaccharide (APS), berberine (BBR) and pueraria flavonoid (PF) that were extracted from traditional Chinese medicines were used to treat diabetic liver, according to which, fragility of glycogen structures could be successfully repaired [42]. Recently, Liu et al. [43] also showed that fragility of diabetic liver glycogen structure could be restored by using two anti-diabetic drugs, that is, metformin and berberine, respectively. It was suggested that the main mechanism of glycogen structure conversion from fragile state to stable state was due to the reduced glycogen phosphorylase (GP) level via the cAMP/PKA signalling pathway and decreased GP affinity with glycogen particles in diabetic livers [43]. In this study, we confirmed that the ethanol extract of mulberry leaves could repair structural fragility of glycogen particles (Fig. 5). Although we did not perform experimental studies in terms of the molecular mechanisms, we noticed that blood glucose level was tightly correlated with glycogen structure by comparing these studies. In particular, higher blood glucose level tends to be associated with fragile glycogen structure while lower blood glucose level seems to be related with stable glycogen structure. However, due to the complex regulation of glycogen metabolism, further experimental studies are required to explore the specific molecular mechanisms in future studies.

Diabetes mellitus is a complex disease with chronic hyperglycemia, which poses serious public health risks worldwide. In this study, ethanol extract of mulberry leaves showed positive effects on reducing the levels of fasting blood glucose, triglycerides, total cholesterol, and low-density lipoproteins while the level of high-density lipoprotein was significantly improved. However, response to glucose was only slightly increased in the MLE group when compared with T2DM group. The bioactive components in mulberry leaves, such as polysaccharides, polyphenols, flavonoids and alkaloids, might be responsible for these beneficial effects, which is worth of further investigation. As for the composition of intestinal microbiota, the abundance of the phyla *Bacteroidetes* and *Proteobacteria* were increased while that of the phylum *Actinobacteria* was reduced. At genus level, both the levels of *Bifidobacterium* and *Lactobacillus* were reduced while the level of *Ruminococcus2* was increased after the treatment of mulberry leaves ethanol extract. In addition, fragility of liver glycogen structure was restored in MLE group, which might be due to the reduced blood glucose level that was modulated by both bioactive components in MLs and the altered composition of microbiota. However, more studies are needed to unravel the molecular mechanisms behind this phenotype change. In sum, our findings support the therapeutic effects of the ethanol extract of mulberry leaves on type 2 diabetes while the exact molecular mechanisms of how the ethanol extract of mulberry leaves alleviates diabetes remains to be investigated.

## Conclusion

Mulberry leaves have been widely used in Asia as a traditional medicine to treat type 2 diabetes mellitus for a long term. Previous studies support its roles in lowering blood glucose level and modulating intestinal microbiota. Recent studies suggested that structural fragility of liver glycogen particles might be a physio-pathological marker for both type 1 and type 2 diabetes mellitus at molecular level, which was probably due to the high blood glucose level. In addition, a couple of studies also discovered that both traditional and western medicine could be able to restore liver glycogen fragility via reducing blood glucose level. However, the effects of mulberry leaves on the structural fragility of liver glycogen particles have never been investigated. In this study, we analyzed the components of the ethanol extract of mulberry leaves, which was then administrated to a group of in-house constructed HFD/STZ-induced type 2 diabetic rats. The effects of the ethanol extract of mulberry leaves on both intestinal microbiota and liver glycogen structure were investigated. The results suggested that ethanol extract of mulberry leaves consisted of multiple bio-active components, the administration of which could restore liver glycogen fragility at molecular level and also altered intestinal microbiota composition. Although some bacterial genera have been linked with the development of diabetes mellitus, whether specific groups of bacteria could influence liver glycogen structure via modulating blood glucose level or other unknown pathways is currently unknown and is worthy of further investigation. Future studies should focus on the molecular mechanisms of anti-diabetic effects of the ethanol extract of mulberry leaves from the aspects of intestinal microbiota modulation and liver glycogen structure restoration.

## Supplementary Information

**Additional file 1.**

**Additional file 2.**

**Additional file 3.**

## Data Availability

All data analysed during this study are included in this published article. The datasets generated during this study are not publicly available but are available from the corresponding author on reasonable request.

## Abbreviations

APS: Astragalus polysaccharide
AUD: Areas under curve
BBR: Berberine
BW: Body weights
FBG: Fasting blood glucose
GOPOD: Glucose oxidase/peroxidase
GP: Glycogen phosphorylase
HDL: High-density lipoprotein
HFD: High-fat diet
HPLC: High performance liquid chromatography
HSD: Honestly significant difference
LC/MS: Liquid chromatography/mass spectrometry
LDL: Low-density lipoprotein
MLE: Mulberry leaves ethanol extract
NC: Normal group
OGTT: Oral glucose tolerance test
OTUs: Operational taxonomic units
PCoA: Principal component analysis
PF: Pueraria flavonoid
SD: Sprague-Dawley
SEC: Size exclusion chromatography
SEM: Standard error of the mean
SPF: Specific pathogen free
STD: Standard deviation
STZ: Streptozotocin
T2DM: Type 2 diabetes mellitus
TC: Total cholesterol
TCM: Traditional Chinese medicine
TG: Triglyceride
