# Supplementary material for: Ethanol extract of mulberry leaves partially restores the composition of intestinal microbiota and strengthens liver glycogen fragility in type 2 diabetic rats
Source: BMC Complement Med Ther. 2021 Jun 14;21:172. doi: 10.1186/s12906-021-03342-x (PMC8204513; doi:10.1186/s12906-021-03342-x)
Supplement: Supplementary file 1 — Additional file 1. [file 12906_2021_3342_MOESM1_ESM.docx]

**Title**

Ethanol extract of mulberry leaves partially restores the composition of intestinal microbiota and strengthens liver glycogen fragility in type 2 diabetic rats

**Running Title**

Mulberry leaf extract and diabetes

Zhan-Zhong Liu^1,2,3,#^, Qing-Hua Liu^2,3,#^, Zhao Liu^4,#^, Jia-Wei Tang^5^, Eng-Guan Chua^6^, Fen Li^7^, Xuesong Xiong^7^, Meng-Meng Wang^2,3^, Peng-Bo Wen^5^, Xin-Yi Shi^8^, Xiang-Yu Xi^1^, Xiao Zhang^5,9,*^, Liang Wang^2,5,10,*^

**Supplementary Table 1** A complete list of all the components detected by LC/MS technique in the ethanol extract of mulberry leaves.

| **No.** | **Components** | **Formula** | **Observed m/z Ratio** | **Observed Retention Time**  **(min)** | **Response** | **Mass Error**  **(mDa)** | **Adduct** |
| --- | --- | --- | --- | --- | --- | --- | --- |
| 1 | Morusimic acid A | C_24_H_45_NO_9_ | 492.3168 | 3.26 | 1162818 | 0.1 | +H |
| 2 | Butyl isobutyl phthalate | C_16_H_22_O_4_ | 301.1414 | 7.48 | 904857 | 0.4 | +Na, +H |
| 3 | Nigakihemiacetal F | C_22_H_32_O_6_ | 415.2118 | 5.95 | 643627 | 2.7 | +Na |
| 4 | Pyrophaeophorbide A | C_33_H_34_N_4_O_3_ | 535.2702 | 9.79 | 643616 | -0.2 | +H |
| 5 | Stearidonic acid | C_18_H_28_O_2_ | 277.2166 | 6.48 | 624786 | 0.4 | +H |
| 6 | Morusimic acid B | C_18_H_35_NO_4_ | 330.2641 | 3.37 | 596700 | 0.2 | +H |
| 7 | Maglifloenone (Denudatone) | C_22_H_26_O_6_ | 387.1802 | 5.41 | 576257 | -0.1 | +H, +Na |
| 8 | Azedarachin C | C_32_H_42_O_10_ | 609.2699 | 9.08 | 507880 | 2.9 | +Na |
| 9 | 13-Hydroxy-9,11-hexadecadienoic acid | C_16_H_28_O_3_ | 291.1957 | 4.97 | 358302 | 2.7 | +Na |
| 10 | Bilirubin | C_33_H_36_N_4_O_6_ | 607.2554 | 9.68 | 301103 | 2.7 | +Na |
| 11 | 5-Ene-methylcholate-3-O-β-D-glucuronopyranosyl-(1→4)-α-L-rhamnopyranoside | C_37_H_58_O_15_ | 743.3825 | 8.26 | 255202 | -2.3 | +H |
| 12 | Cuscohygrine | C_13_H_24_N_20_ | 225.1965 | 5.06 | 248375 | 0.4 | +H |
| 13 | 14-Deoxy-11-hydro-xyandrographolide | C_20_H_30_O_5_ | 351.2140 | 4.07 | 204526 | -2.6 | +H |
| 14 | 3-O-[β-D-Glucopyra-nosyl-(1→2)]-β-D-glucopyranosyl-kaempferol | C_27_H_30_O_16_ | 611.1604 | 2.74 | 163757 | -0.2 | +H, +Na |
| 15 | Morin | C_15_H_10_O_7_ | 303.0499 | 2.87 | 163264 | 0 | +H |
| 16 | 6-Hydroxykaempferol-3-O-glucoside | C_21_H_20_O_12_ | 465.1024 | 2.87 | 155741 | -0.4 | +H, +Na |
| 17 | Meso-dihydroguaiaretic acid | C_20_H_26_O_4_ | 331.1878 | 4.97 | 147185 | -2.6 | +H |
| 18 | 7-Hydroxy-1-methoxy-2-methoxyxanthone | C_15_H_10_O_6_ | 287.0549 | 3.15 | 136569 | -0.2 | +H |
| 19 | Sucrose | C_12_H_22_O_11_ | 365.1051 | 0.51 | 132716 | -0.4 | +Na, +H |
| 20 | Kaempferol 3-O-β-D-glucopyranoside | C_21_H_20_O_11_ | 449.1072 | 3.15 | 131456 | -0.6 | +H, +Na |
| 21 | Paeonisothujone | C_10_H_14_O_3_ | 205.0858 | 7.49 | 127669 | 2.2 | +Na |
| 22 | Saurufuran A | C_20_H_28_O_3_ | 317.2088 | 6.53 | 126396 | -2.3 | +H |
| 23 | N-Benzoylphenylalanyl-L-phenylalaninol acetate | C_27_H_28_N_2_O_4_ | 445.2119 | 5.81 | 125277 | -0.3 | +H, +Na |
| 24 | Digiprolactone | C_11_H_16_O_3_ | 197.1173 | 0.93 | 118107 | 0 | +H |
| 25 | Bis(2-ethylhexyl)phthalate | C_24_H_38_O_4_ | 391.2842 | 9.99 | 112083 | -0.1 | +H, +Na |
| 26 | 10-O-Methyl alismoxide | C_16_H_28_O_2_ | 275.2008 | 4.07 | 110718 | 2.6 | +Na |
| 27 | Pingpeimine B | C_27_H_45_NO_6_ | 502.3166 | 8.25 | 107191 | 2.7 | +Na |
| 28 | Oxyphyllacinol | C_20_H_26_O_3_ | 315.1932 | 6.83 | 106290 | -2.3 | +H |
| 29 | 2-Monolinolein | C_21_H_38_O_4_ | 355.2818 | 8.55 | 104503 | -2.5 | +H |
| 30 | Nigakilactone H | C_22_H_32_O_8_ | 425.2146 | 8.06 | 104361 | -2.4 | +H |
| 31 | Physalin G | C_28_H_30_O_10_ | 549.1720 | 4.72 | 101584 | -1.1 | +Na |
| 32 | Ajugaside A | C_32_H_50_O_14_ | 659.3245 | 6.58 | 98307 | -2.8 | +H |
| 33 | 4,7-Didehydroneophysalin B | C_28_H_28_O_9_ | 509.1804 | 5.17 | 97897 | -0.2 | +H, +Na |
| 34 | Ciwujiatone | C_22_H_26_O_9_ | 435.1665 | 4.58 | 97507 | 1.5 | +H |
| 35 | Trichosanic acid | C_18_H_30_O_2_ | 279.2321 | 6.97 | 97201 | 0.3 | +H |
| 36 | Daturametelin E | C_29_H_42_O_10_S | 605.2387 | 8.78 | 95134 | -0.4 | +Na |
| 37 | Dihydro-N-methylisopelletierine | C_9_H_19_NO | 158.1538 | 4.95 | 89103 | -0.1 | +H |
| 38 | Delbruline | C_26_H_41_NO_7_ | 502.2802 | 5.95 | 84634 | 2.7 | +Na |
| 39 | 2-Benzyl octanal | C_15_H_22_O | 219.1745 | 6.10 | 82346 | 0.1 | +H |
| 40 | Ilexin Ⅱ | C_23_H_30_O_10_ | 467.1938 | 5.81 | 80501 | 2.7 | +H |
| 41 | Phthalic anhydride | C_8_H_4_O_3_ | 149.0234 | 7.48 | 79125 | 0 | +H |
| 42 | 5-Ene-methylcholate-3-O-β-D-glucuronopyranosyl-(1→4)-α-L-rhamnopyranoside | C_37_H_58_O_15_ | 743.3822 | 7.88 | 76890 | -2.7 | +H |
| 43 | 1,7-Bis(4-hydroxyphenyl)-hepta-4E,6E-dien-3-one | C_19_H_18_O_3_ | 317.1149 | 0.82 | 76212 | 0.1 | +Na |
| 44 | 11-O-p-Coumarylnepeticin | C_39_H_56_O_4_ | 589.4278 | 8.93 | 70281 | 2.7 | +H |
| 45 | Kosamol A | C_30_H_38_O_8_ | 549.2484 | 9.05 | 68261 | 2.5 | +Na |
| 46 | 2-((3R,4R)-7-Hydroxy-4-(4-hydroxy-5-((R)-7-hydroxychroman-3-yl)-2-  methoxyphenyl)chroman-3-yl)-5-methoxycyclohexa-2,5-diene-1,4-dione | C_32_H_28_O_9_ | 557.1792 | 4.99 | 67582 | -1.4 | +H, +Na |
| 47 | 9,16-Dioxyhydroxy-10,12,14-triene-18 carbonic acid | C_18_H_30_O_4_ | 333.2026 | 5.75 | 66849 | -1 | +Na |
| 48 | Dihydroactinidiolide | C_11_H_16_O_2_ | 181.1223 | 4.85 | 66441 | 0 | +H |
| 49 | 14-epi-Andrographolide | C_20_H_30_O_5_ | 351.2142 | 4.34 | 64874 | -2.4 | +H |
| 50 | Genistein-7,4'-di-O-β-D-glucoside | C_27_H_30_O_15_ | 595.1649 | 3.02 | 63103 | -0.8 | +H, +Na |
| 51 | Dendronobilin B | C_15_H_24_O_5_ | 285.1672 | 7.48 | 59581 | -2.5 | +H |
| 52 | Daturametelin B | C_34_H_48_O_10_ | 639.3149 | 9.37 | 58758 | 1 | +Na |
| 53 | 7β-Angeloyloxyoplopa-3(14)Z,8(10)-dien-2-one | C_20_H_28_O_3_ | 317.2090 | 7.34 | 56903 | -2.1 | +H |
| 54 | Bufotalinin | C_24_H_30_O_6_ | 437.1936 | 6.08 | 55240 | 0.1 | +Na |
| 55 | Linolenic acid | C_18_H_30_O_2_ | 279.2317 | 8.49 | 54883 | -0.1 | +H |
| 56 | Sanleng acid | C_18_H_34_O_5_ | 353.2293 | 4.33 | 54249 | -0.5 | +Na |
| 57 | 14-Deoxyandrographolide | C_20_H_30_O_4_ | 335.2188 | 5.55 | 47497 | -2.9 | +H |
| 58 | (10E)1,10-Heptadeca-diene-4,6-diyne-3,8,9-triol | C_17_H_24_O_3_ | 277.1799 | 5.28 | 45944 | 0.1 | +H |
| 59 | α-Estradiol | C_18_H_24_O_2_ | 273.1846 | 4.97 | 45682 | -0.3 | +H |
| 60 | 7β-Angeloyloxyoplopa-3(14)Z,8(10)-dien-2-one | C_20_H_28_O_3_ | 317.2087 | 7.46 | 44953 | -2.5 | +H |
| 61 | Kadsurenin J | C_23_H_28_O_6_ | 423.1798 | 6.09 | 43490 | 2 | +Na |
| 62 | Corypalline | C_11_H_15_NO_2_ | 194.1174 | 5.81 | 42108 | -0.2 | +H |
| 63 | Bistortaside | C_22_H_24_O_14_ | 535.1070 | 2.64 | 41512 | 1.2 | +Na |
| 64 | Delbrusine | C_27_H_43_NO_7_ | 516.2958 | 5.94 | 41478 | 2.6 | +Na |
| 65 | 2β-(Isobutryloxy) florenalin | C_19_H_26_O_5_ | 335.1828 | 5.28 | 41179 | -2.5 | +H |
| 66 | Chenodeoxycholic acid | C_24_H_40_O_4_ | 393.2978 | 9.88 | 38420 | -2.2 | +H |
| 67 | Herbacetin | C_15_H_10_O_7_ | 303.0498 | 2.72 | 37822 | -0.1 | +H |
| 68 | Sanleng acid | C_18_H_34_O_5_ | 353.2293 | 4.68 | 37586 | -0.5 | +Na |
| 69 | 2-Methoxybenzyl-2,3,6-trimethoxybenzoate | C_18_H_20_O_6_ | 333.1356 | 6.47 | 37440 | 2.4 | +H |
| 70 | Glehlinoside A | C_34_H_42_O_14_ | 675.2644 | 3.98 | 37429 | -0.3 | +H, +Na |
| 71 | Jangomolide | C_26_H_28_O_8_ | 491.1699 | 5.17 | 36833 | 2.3 | +Na |
| 72 | Astragaline F | C_12_H_18_N_2_O_5_ | 293.1090 | 5.06 | 36176 | -1.8 | +Na |
| 73 | Heptadecylamine | C_17_H_37_N | 256.3000 | 7.72 | 36019 | 0.1 | +H |
| 74 | Foresticine | C_24_H_39_NO_6_ | 460.2697 | 5.95 | 35348 | 2.8 | +Na |
| 75 | Tenuifoliside D | C_18_H_24_O_9_ | 385.1510 | 4.07 | 34481 | 1.7 | +H |
| 76 | Nigellamose | C_18_H_32_O_16_ | 527.1574 | 0.50 | 34005 | -0.8 | +Na |
| 77 | Fuzinoside | C_15_H_28_O_13_ | 439.1417 | 0.49 | 32197 | -0.5 | +Na |
| 78 | Lucidenic acid D2 methyl ester | C_30_H_40_O_8_ | 551.2633 | 9.63 | 30626 | 1.7 | +Na |
| 79 | 6α-Acetoxy-5-epilimonin | C_30_H_38_O_9_ | 565.2425 | 9.83 | 29238 | 1.7 | +Na |
| 80 | 9,16-Dioxyhydroxy-10,12,14-triene-18 carbonic acid | C_18_H_30_O_4_ | 311.2203 | 4.06 | 28768 | -1.4 | +H |
| 81 | Oriediterpenol | C_20_H_32_O_2_ | 305.2474 | 8.04 | 27061 | -0.1 | +H |
| 82 | Lobelanidine | C_22_H_29_NO_2_ | 340.2297 | 6.04 | 26672 | 2.6 | +H |
| 83 | Bletilol B | C_27_H_26_O_7_ | 463.1746 | 5.17 | 26549 | -0.5 | +H |
| 84 | Eugenyl glucoside | C_16_H_22_O_7_ | 349.1255 | 3.40 | 25690 | -0.3 | +Na |
| 85 | Dihydrocostunolide | C_15_H_22_O_2_ | 235.1689 | 4.81 | 24824 | -0.3 | +H |
| 86 | (+)-Syringaresinol-O-β-D-glucopyranoside | C_28_H_36_O_13_ | 603.2035 | 3.05 | 24350 | -1.3 | +Na |
| 87 | Demethylwedelolactone | C_15_H_8_O_7_ | 301.0337 | 2.87 | 24321 | -0.6 | +H |
| 88 | 1-Deoxynojirimycin (DNJ) | C_6_H_13_NO_4_ | 164.0914 | 0.69 | 23030 | -0.3 | +H |
| 89 | Lignoceryl ferulate | C_34_H_58_O_4_ | 553.4246 | 6.47 | 22769 | 1.8 | +Na |
| 90 | (E,E)-9-Oxooctadeca-10,12-dienoic acid | C_18_H_30_O_3_ | 295.2265 | 4.33 | 22759 | -0.3 | +H |
| 91 | Oriediterpenoside | C_25_H_40_O_6_ | 437.2899 | 3.49 | 22736 | 0.2 | +H |
| 92 | Morusimic acid E | C_24_H_45_NO_10_ | 508.3107 | 3.00 | 22471 | -0.9 | +H |
| 93 | N-cis-Feruloyl typamine | C_18_H_19_NO_4_ | 314.1382 | 3.71 | 22046 | -0.5 | +H |
| 94 | Panaxytriol | C_17_H_26_O_3_ | 279.1957 | 6.06 | 21995 | 0.3 | +H |
| 95 | 5,7,2',5'-Tetrahydroxy-flavone | C_15_H_10_O_6_ | 287.0546 | 2.99 | 21723 | -0.4 | +H |
| 96 | Quercetin-3-O-α-L-rhamnoside | C_21_H_20_O_11_ | 449.1071 | 2.92 | 21678 | -0.7 | +H |
| 97 | p-Methoxybenzylacetone | C_11_H_14_O_2_ | 179.1063 | 0.93 | 21670 | -0.4 | +H |
| 98 | Blumenol C glucoside | C_19_H_32_O_7_ | 373.2211 | 3.12 | 20818 | -1 | +H, +Na |
| 99 | Xanthorrhizol | C_15_H_22_O | 219.1742 | 6.81 | 20798 | -0.2 | +H |
| 100 | β-D-Fructose | C_6_H_12_O_6_ | 203.0521 | 0.51 | 20374 | -0.6 | +Na |
| 101 | Blestrianol D | C_29_H_24_O_5_ | 453.1674 | 5.94 | 19624 | -2.3 | +H |
| 102 | Neoline | C_24_H_39_NO_6_ | 460.2695 | 3.13 | 19062 | 2.6 | +Na |
| 103 | Picrasin G | C_21_H_28_O_7_ | 393.1881 | 2.93 | 18651 | -2.7 | +H |
| 104 | Methyl lucidenate P | C_30_H_44_O_8_ | 555.2921 | 5.75 | 18271 | -0.8 | +Na |
| 105 | Maokonine | C_12_H_17_NO_3_ | 224.1276 | 4.89 | 17729 | -0.5 | +H |
| 106 | Indigotin | C_16_H_10_N_2_O_2_ | 285.0619 | 3.99 | 16637 | -1.5 | +Na |
| 107 | Evodin | C_26_H_30_O_8_ | 493.1839 | 4.66 | 16370 | 0.6 | +Na |
| 108 | Licoricone | C_22_H_22_O_6_ | 383.1501 | 4.07 | 15645 | 1.2 | +H |
| 109 | Ingenol-20-hexadecanoate | C_36_H_58_O_6_ | 609.4104 | 9.62 | 15251 | -2.2 | +Na |
| 110 | Asterinin D | C_25_H_33_N_5_O_7_ | 538.2271 | 2.70 | 14658 | -0.1 | +Na |
| 111 | Coronaric acid | C_18_H_32_O_3_ | 319.2239 | 7.80 | 14656 | -0.4 | +Na |
| 112 | Ent-eudesmane-2α,4β,11-triol 11-O-β-D-glucopyranoside | C_21_H_38_O_8_ | 441.2440 | 3.27 | 14585 | -1.9 | +Na |
| 113 | Vitetrifolin E | C_22_H_36_O_4_ | 365.2672 | 8.80 | 14509 | -1.4 | +H, +Na |
| 114 | Rengyoside C | C_22_H_32_O_10_ | 479.1895 | 3.80 | 14427 | 0.7 | +Na |
| 115 | Rhodojaponin Ⅵ | C_20_H_34_O_7_ | 387.2362 | 5.47 | 14218 | -1.6 | +H |
| 116 | Ophiopogonanone B | C_18_H_18_O_5_ | 337.1040 | 6.30 | 14178 | -0.6 | +Na |
| 117 | 2,7-Dihydroxy-1-(4'-hydroxybenzyl)-4-methoxy-9,10-dihydrophenanthrene-4'-O-  glucoside | C_28_H_30_O_9_ | 511.1941 | 5.80 | 13930 | -2.2 | +H |
| 118 | 6-Gingerol | C_17_H_26_O_4_ | 295.1893 | 5.28 | 13841 | -1.1 | +H |
| 119 | 13-Hydroxygermacrone | C_15_H_22_O_2_ | 235.1688 | 4.14 | 13744 | -0.5 | +H |
| 120 | Darutigenol | C_20_H_34_O_3_ | 345.2391 | 8.04 | 13620 | -0.9 | +Na |
| 121 | Maltol | C_6_H_6_O_3_ | 149.0227 | 0.63 | 13482 | 1.8 | +Na |
| 122 | Lucidenic acid A | C_27_H_38_O_6_ | 481.2586 | 2.86 | 13454 | 2.5 | +Na |
| 123 | Coronaric acid | C_18_H_32_O_3_ | 297.2397 | 8.22 | 13428 | -2.7 | +H |
| 124 | 12S-Hydroxyandrographolide | C_20_H_32_O_6_ | 369.2262 | 8.05 | 13360 | -1 | +H |
| 125 | Paristerone | C_27_H_44_O_7_ | 481.3159 | 3.56 | 13335 | -0.1 | +H |
| 126 | 1-Methyl-2-[(6Z,9Z)-6,9-pentadecadienyl]-4(1H)-quinolone | C_25_H_35_NO | 388.2631 | 3.02 | 13212 | 2 | +Na |
| 127 | Eclalbasaponin Ⅴ | C_36_H_58_O_12_S | 715.3734 | 4.06 | 13151 | 1.3 | +H |
| 128 | Periplocoside O | C_36_H_56_O_10_ | 671.3792 | 8.69 | 13076 | 2.6 | +Na |
| 129 | Melianol | C_35_H_48_O_9_ | 635.3213 | 10.15 | 12994 | 2.2 | +Na |
| 130 | Nobilin C | C_18_H_22_O_6_ | 335.1506 | 6.97 | 12743 | 1.7 | +H |
| 131 | 2-O-α-D-Glycosides of galactose-1-deoxynojirimycin | C_12_H_23_NO_9_ | 326.1436 | 0.66 | 12702 | -1 | +H |
| 132 | Cynanoside Q2 | C_40_H_60_O_14_ | 787.3857 | 8.95 | 12462 | -1.9 | +Na |
| 133 | 12S-Hydroxyandrographolide | C_20_H_32_O_6_ | 391.2068 | 5.26 | 12317 | -2.3 | +Na |
| 134 | Hexadecanoic acid | C_16_H_32_O_2_ | 279.2285 | 6.57 | 11808 | -1 | +Na |
| 135 | 2′-Hydroxy-4,4′,6′-trimethoxydihydrochalcone | C_18_H_20_O_5_ | 317.1371 | 3.80 | 11796 | -1.2 | +H |
| 136 | Blestriarene B | C_30_H_24_O_6_ | 481.1661 | 3.11 | 11589 | 1.5 | +H |
| 137 | Acsonine | C_31_H_41_NO_8_ | 556.2935 | 4.84 | 11452 | 3 | +H |
| 138 | 9,12-Dihydroxy-15-nonadecenoic acid | C_19_H_36_O_4_ | 351.2526 | 5.99 | 11342 | 2 | +Na |
| 139 | Tomentogenin | C_21_H_36_O_5_ | 391.2445 | 6.71 | 11299 | -1 | +Na |
| 140 | 12-α-Hydroxylimonin | C_26_H_30_O_9_ | 509.1787 | 3.98 | 11232 | 0.5 | +Na |
| 141 | Demethyl auraptenol | C_14_H_14_O_4_ | 269.0804 | 4.84 | 11230 | 2 | +Na |
| 142 | Catenarin | C_15_H_10_O_6_ | 287.0540 | 2.64 | 11061 | -1 | +H |
| 143 | Coniferol | C_10_H_12_O_3_ | 181.0860 | 3.80 | 11037 | 0.1 | +H |
| 144 | Isolappaol C | C_30_H_34_O_10_ | 577.2036 | 5.63 | 11037 | -0.8 | +Na |
| 145 | Oxyphyllenone A | C_12_H_18_O_3_ | 211.1323 | 3.95 | 11002 | -0.6 | +H |
| 146 | Proline | C_5_H_9_NO_2_ | 138.0543 | 0.51 | 10694 | 1.8 | +Na |
| 147 | Heterodendrin | C_11_H_19_NO_6_ | 262.1281 | 0.52 | 10686 | -0.4 | +H |
| 148 | n-Nonyl acetate | C_11_H_22_O_2_ | 209.1533 | 2.92 | 10648 | 2.1 | +Na |
| 149 | Albiflorin | C_23_H_28_O_11_ | 481.1676 | 3.26 | 10608 | -2.8 | +H |
| 150 | Dibutyl sebacate | C_18_H_34_O_4_ | 337.2340 | 8.86 | 10496 | -1 | +Na |
| 151 | Cnidilide | C_12_H_18_O_2_ | 195.1374 | 4.07 | 10482 | -0.6 | +H |
| 152 | Thymol isobutyrate | C_14_H_20_O_2_ | 243.1356 | 6.79 | 10370 | 0 | +Na |
| 153 | Kaempferol | C_15_H_10_O_6_ | 287.0553 | 4.00 | 10364 | 0.3 | +H |
| 154 | Picrasinoside H | C_30_H_44_O_13_ | 613.2841 | 4.06 | 10364 | -1.4 | +H |
| 155 | Safrol | C_10_H_10_O_2_ | 163.0753 | 3.80 | 10358 | 0 | +H |
| 156 | AH20 | C_51_H_46_O_14_ | 883.2974 | 8.26 | 10298 | 1.4 | +H |
| 157 | Scutellone I | C_28_H_36_O_7_ | 507.2347 | 5.21 | 10285 | -0.6 | +Na |
| 158 | Fagomine | C_6_H_13_NO_3_ | 148.0961 | 0.74 | 10216 | -0.8 | +H |
| 159 | Zederone | C_15_H_18_O_3_ | 247.1317 | 2.82 | 10125 | -1.1 | +H |
| 160 | 3,7-Dimethyloctane-1,3,6-triol | C_10_H_22_O_3_ | 213.1470 | 4.07 | 9892 | 0.9 | +Na |
| 161 | 2′-Hydroxy-7,3′,4′-trimethoxy-isoflavan | C_18_H_20_O_5_ | 317.1380 | 3.61 | 9739 | -0.3 | +H |
| 162 | Picrasinol B | C_22_H_32_O_6_ | 415.2100 | 6.24 | 9734 | 0.9 | +Na |
| 163 | Deoxypaeonisuffrone | C_10_H_14_O_3_ | 205.0855 | 3.06 | 9516 | 2 | +Na |
| 164 | Salsoline | C_11_H_15_NO_2_ | 194.1170 | 0.55 | 9485 | -0.6 | +H |
| 165 | Glycocholic acid | C_26_H_43_NO_6_ | 488.3011 | 5.95 | 9339 | 2.9 | +Na |
| 166 | ent-Kauran-16α,17-diol | C_20_H_34_O_2_ | 307.2628 | 8.47 | 9332 | -0.4 | +H |
| 167 | Tribulusamide A | C_36_H_36_N_2_O_8_ | 625.2517 | 4.65 | 9083 | -2.8 | +H |
| 168 | Campesterol acetate | C_30_H_50_O_2_ | 465.3723 | 9.93 | 9052 | 2 | +Na |
| 169 | 19β-Glucosyl-14-deoxyandrographoside | C_26_H_40_O_9_ | 497.2757 | 6.79 | 8990 | 1.2 | +H |
| 170 | Cimidahuside C | C_37_H_58_O_12_ | 695.4015 | 4.06 | 8956 | 1.4 | +H |
| 171 | Ganoderenic acid B | C_30_H_42_O_7_ | 515.3022 | 3.50 | 8856 | 1.9 | +H |
| 172 | Baicalein-7-O-β-D glucopyranoside | C_21_H_20_O_10_ | 433.1112 | 3.23 | 8850 | -1.7 | +H |
| 173 | Astin E | C_25_H_32_ClN_57_ | 572.1876 | 5.17 | 8439 | -0.7 | +Na |
| 174 | Andrograpanin | C_20_H_30_O_3_ | 319.2255 | 6.49 | 8407 | -1.3 | +H |
| 175 | Kihadanin B | C_26_H_30_O_9_ | 509.1789 | 4.35 | 8403 | 0.7 | +Na |
| 176 | Shanciol F | C_25_H_24_O_6_ | 421.1636 | 7.21 | 8388 | -1 | +H |
| 177 | Ginkgol | C_21_H_34_O | 325.2479 | 4.13 | 8377 | -2.2 | +Na |
| 178 | 6-Feruloyl catalpol | C_25_H_30_O_12_ | 545.1614 | 3.90 | 8345 | -1.5 | +Na |
| 179 | 1-Methoxy-3,7-dimethyl-2,6-octadiene | C_11_H_20_O | 191.1428 | 2.92 | 8202 | 2.1 | +Na |
| 180 | Delphatine | C_26_H_43_NO_7_ | 504.2951 | 2.85 | 8198 | 2 | +Na |
| 181 | Pterosin Y | C_15_H_20_O_5_ | 281.1374 | 5.94 | 8193 | -1 | +H |
| 182 | Urolignoside | C_26_H_34_O_11_ | 545.1980 | 2.64 | 8146 | -1.3 | +Na |
| 183 | Blumenol C glucoside | C_19_H_32_O_7_ | 395.2034 | 2.39 | 8137 | -0.6 | +Na |
| 184 | 2,3,5,4'-Tetrahydroxystilbene-2-O-β-D-glucopyranoside | C_20_H_22_O_9_ | 429.1145 | 3.47 | 8114 | -1.1 | +Na |
| 185 | 2,7-Dihydroxy-4-methoxyphenanthrene-2-O-glucoside | C_21_H_22_O_8_ | 403.1386 | 5.18 | 8037 | -0.1 | +H |
| 186 | Florilenalin angelate | C_20_H_26_O_5_ | 369.1698 | 5.41 | 7981 | 2.5 | +Na |
| 187 | Bigelovin | C_17_H_20_O_5_ | 327.1207 | 4.65 | 7916 | 0.4 | +Na |
| 188 | Lupinifolin | C_25_H_26_O_5_ | 407.1846 | 7.45 | 7911 | -0.7 | +H |
| 189 | epi-Kansenone | C_30_H_48_O_2_ | 441.3697 | 10.09 | 7871 | -3 | +H |
| 190 | Glycerol-β-steariate | C_21_H_42_O_4_ | 359.3150 | 9.83 | 7848 | -0.6 | +H |
| 191 | (S)-Shihulimonin A | C_26_H_30_O_10_ | 503.1900 | 3.17 | 7724 | -1.2 | +H |
| 192 | (+)-Bakuchiol | C_18_H_24_O | 257.1891 | 4.07 | 7308 | -0.9 | +H |
| 193 | Nobilin D | C_16_H_18_O_6_ | 307.1189 | 7.48 | 7233 | 1.3 | +H |
| 194 | Ranunculin | C_11_H_16_O_8_ | 277.0935 | 0.51 | 7198 | 1.7 | +H |
| 195 | Daturametelin A | C_34_H_48_O_9_ | 601.3383 | 6.22 | 7068 | 1.2 | +H |
| 196 | BaohuosideⅠ | C_27_H_30_O_10_ | 515.1884 | 4.19 | 6968 | -2.8 | +H |
| 197 | Isoxanthanol | C_17_H_24_O_5_ | 331.1533 | 2.54 | 6921 | 1.7 | +Na |
| 198 | Asterinin C | C_26_H_35_N_5_O_8_ | 568.2376 | 3.10 | 6890 | -0.2 | +Na |
| 199 | Morachalcone A | C_20_H_20_O_5_ | 341.1363 | 4.77 | 6750 | -2.1 | +H |
| 200 | Phenethyl ferulate | C_18_H_18_O_4_ | 299.1268 | 3.80 | 6577 | -0.9 | +H |
| 201 | (25S)-5β-Spirostan-3β-ol-3-O-α-L-rhamnop-yranosyl(1→2)-[β-D-glucopyranosyl  (1→4)]-β-D-galactopyranoside | C_45_H_74_O_17_ | 887.4979 | 8.45 | 6577 | -2 | +H |
| 202 | Thymol isobutyrate | C_14_H_20_O_2_ | 221.1535 | 5.27 | 6547 | -0.1 | +H |
| 203 | (3R)-Abruquinone B | C_20_H_22_O_8_ | 413.1192 | 4.15 | 6475 | -1.5 | +Na |
| 204 | Bufotalin | C_26_H_36_O_6_ | 467.2424 | 5.21 | 6318 | 1.9 | +Na |
| 205 | Ginkgolic acid | C_22_H_34_O_3_ | 347.2559 | 8.47 | 6298 | -2.2 | +H |
| 206 | Eugenol | C_10_H_12_O_2_ | 165.0911 | 3.40 | 6254 | 0 | +H |
| 207 | 14(R)-Hydroxy-7β-isovaleroyloxyoplop-8(10)-en-2-one | C_20_H_32_O_4_ | 359.2183 | 6.49 | 6193 | -1 | +Na |
| 208 | Burchellin | C_20_H_20_O_5_ | 341.1372 | 5.89 | 6081 | -1.1 | +H |
| 209 | Fibraurin | C_20_H_20_O_7_ | 373.1272 | 5.60 | 6036 | -1 | +H |
| 210 | Eclalbasaponin ⅩⅢ | C_37_H_58_O_10_ | 685.3943 | 3.33 | 5829 | 2.1 | +Na |
| 211 | Rubilactone | C_15_H_10_O_5_ | 271.0597 | 4.37 | 5770 | -0.4 | +H |
| 212 | N-Isobutyl-(2E,4E)-octadecadienamide | C_22_H_41_NO | 336.3247 | 9.72 | 5710 | -1.4 | +H |
| 213 | Sesquipinsapol B | C_30_H_36_O_9_ | 563.2269 | 9.56 | 5700 | 1.8 | +Na |
| 214 | Chloranoside A | C_21_H_28_O_9_ | 425.1818 | 5.12 | 5690 | 1.2 | +H |
| 215 | Isosamarcandin | C_29_H_38_O_6_ | 505.2554 | 5.91 | 5675 | -0.6 | +Na |
| 216 | 1-Deoxyeucommiol | C_9_H_16_O_3_ | 173.1163 | 4.06 | 5646 | -0.9 | +H |
| 217 | Methyl arteannuate | C_16_H_24_O_2_ | 271.1687 | 5.37 | 5462 | 1.9 | +Na |
| 218 | Chrysoeriol-7-O-β-D-glucuroside | C_22_H_22_O_11_ | 463.1222 | 3.28 | 5447 | -1.3 | +H |
| 219 | Taurodeoxycholic acid | C_26_H_45_NO_6_S | 522.2875 | 2.74 | 5436 | 1.5 | +Na |
| 220 | 1,2,3-Trimethoxy-5-(2-propenyl)-benzene | C_12_H_16_O_3_ | 209.1166 | 3.67 | 5418 | -0.6 | +H |
| 221 | Lobetyolin | C_20_H_28_O_8_ | 419.1687 | 3.05 | 5418 | 1.1 | +Na |
| 222 | Methyl kushenol C | C_26_H_28_O_7_ | 475.1752 | 0.50 | 5278 | 2.4 | +Na |
| 223 | 2-Monolinolein | C_21_H_38_O_4_ | 377.2634 | 8.49 | 5262 | -2.9 | +Na |
| 224 | Adenine | C_5_H_5_N_5_ | 136.0608 | 0.62 | 5174 | -0.9 | +H |
| 225 | Sanjoinenine | C_29_H_35_N_3_O_4_ | 490.2685 | 5.81 | 5138 | -1.5 | +H |
| 226 | Benzyl benzoate | C_14_H_12_O_2_ | 235.0720 | 0.83 | 5060 | -1 | +Na |
| 227 | 14-Deoxy-12S-methoxyandrographolide | C_21_H_32_O_5_ | 365.2299 | 4.66 | 5037 | -2.4 | +H |
| 228 | Interiotherin C | C_30_H_36_O_10_ | 579.2171 | 5.78 | 5015 | -2.9 | +Na |
| 229 | 2′,4′-Dihydroxy-4,6′-dimethoxy-dihydrochalcone | C_17_H_18_O_5_ | 325.1063 | 5.47 | 4874 | 1.7 | +Na |
| 230 | Picroside Ⅲ | C_25_H_30_O_13_ | 561.1583 | 3.33 | 4808 | 0.4 | +Na |
| 231 | 7β-(3-Ethyl-cis-crotonoyloxy)-14-hydroxynotonipetranone | C_21_H_32_O_4_ | 349.2378 | 3.32 | 4780 | 0.4 | +H |
| 232 | Physalin D | C_28_H_32_O_11_ | 567.1809 | 3.97 | 4733 | -2.7 | +Na |
| 233 | Digiprolactone | C_11_H_16_O_3_ | 219.1011 | 2.96 | 4687 | 2 | +Na |
| 234 | Asterinin B | C_26_H_35_N_5_O_8_ | 568.2354 | 2.79 | 4626 | -2.4 | +Na |
| 235 | Baimuxifuranic acid | C_15_H_24_O_3_ | 253.1790 | 6.39 | 4555 | -0.8 | +H |
| 236 | Aduncin | C_15_H_18_O_6_ | 295.1178 | 5.94 | 4487 | 0.1 | +H |
| 237 | Yakuchinone B | C_20_H_22_O_3_ | 311.1661 | 6.55 | 4411 | 1.9 | +H |
| 238 | Courmaric acid | C_9_H_8_O_2_ | 149.0588 | 3.61 | 4400 | -0.9 | +H |
| 239 | Schininallylol | C_20_H_24_O_5_ | 367.1494 | 6.04 | 4300 | -2.2 | +Na |
| 240 | 3,6-Dihydroxy-p-menth-1-ene | C_11_H_20_O | 191.1430 | 5.96 | 4258 | 2.4 | +Na |
| 241 | Pinocembrin_1 | C_15_H_12_O_4_ | 257.0796 | 3.88 | 4198 | -1.2 | +H |
| 242 | α-Ionone | C_13_H_20_O | 193.1584 | 5.04 | 3900 | -0.3 | +H |
| 243 | N-Benzoyl-phenylalanine-2-benzoylamino-3-phenylpropyl ester | C_32_H_30_N_2_O_4_ | 507.2265 | 6.58 | 3882 | -1.4 | +H |
| 244 | Kushenol M | C_30_H_36_O_7_ | 531.2341 | 5.87 | 3819 | -1.2 | +Na |
| 245 | Lactinolide | C_10_H_16_O_4_ | 223.0955 | 7.44 | 3661 | 1.4 | +Na |
| 246 | Deoxyadenosine | C_10_H_13_N_5_O_3_ | 252.1073 | 0.50 | 3436 | -1.8 | +H |
| 247 | 3-(2'-Carboxyphenyl)-4(3H)-quinazolinone | C_15_H_10_N_2_O_3_ | 289.0565 | 0.50 | 3258 | -1.8 | +Na |
| 248 | 2-Hydroxy-5-methoxy acetophenone | C_9_H_10_O_3_ | 167.0687 | 3.61 | 3133 | -1.6 | +H |
