# Supplementary material for: Ethanol extract of mulberry leaves partially restores the composition of intestinal microbiota and strengthens liver glycogen fragility in type 2 diabetic rats
Source: BMC Complement Med Ther. 2021 Jun 14;21:172. doi: 10.1186/s12906-021-03342-x (PMC8204513; doi:10.1186/s12906-021-03342-x)
Supplement: Supplementary file 2 — Additional file 2. [file 12906_2021_3342_MOESM2_ESM.docx]

**Title**

Ethanol extract of mulberry leaves partially restores the composition of intestinal microbiota and strengthens liver glycogen fragility in type 2 diabetic rats

**Running Title**

Mulberry leaf extract and diabetes

Zhan-Zhong Liu^1,2,3,#^, Qing-Hua Liu^2,3,#^, Zhao Liu^4,#^, Jia-Wei Tang^5^, Eng-Guan Chua^6^, Fen Li^7^, Xuesong Xiong^7^, Meng-Meng Wang^2,3^, Peng-Bo Wen^5^, Xin-Yi Shi^8^, Xiang-Yu Xi^1^, Xiao Zhang^5,9,*^, Liang Wang^2,5,10,*^

**Supplementary Table 2** Composition of intestinal microbiota at phylum level in each sample of the three groups, NC (n=6), T2DM (n=6), and MLE (n=6).

|  | ***Firmicutes*** | ***Bacteroidetes*** | ***Actinobacteria*** | ***Proteobacteria*** | ***Candidatus Saccharibacteria*** | ***unclassified*** | ***Verrucomicrobia*** | ***Tenericutes*** | ***Fusobacteria*** |
| --- | --- | --- | --- | --- | --- | --- | --- | --- | --- |
| **NC-1** | 61.30 | 36.09 | 0.32 | 1.82 | 0.37 | 0.06 | 0.03 | 0.01 | - |
| **NC-2** | 56.62 | 41.62 | 0.30 | 1.13 | 0.28 | 0.02 | - | 0.01 | - |
| **NC-3** | 55.16 | 42.69 | 0.14 | 1.77 | 0.20 | 0.03 | 0.01 | - | - |
| **NC-4** | 56.19 | 42.39 | 0.20 | 1.05 | 0.10 | 0.06 | - | 0.02 | - |
| **NC-5** | 53.71 | 43.99 | 0.16 | 1.87 | 0.21 | 0.04 | - | 0.02 | - |
| **NC-6** | 51.34 | 46.83 | 0.24 | 1.45 | 0.10 | 0.03 | - | 0.01 | - |
| **T2DM-1** | 43.01 | 8.92 | 47.63 | 0.37 | 0.04 | 0.03 | 0.01 | - | - |
| **T2DM-2** | 52.04 | 24.65 | 22.77 | 0.38 | 0.10 | 0.05 | 0.01 | - | - |
| **T2DM-3** | 66.45 | 16.39 | 16.74 | 0.36 | 0.03 | 0.02 | - | - | - |
| **T2DM-4** | 56.28 | 15.68 | 27.50 | 0.23 | 0.12 | 0.02 | 0.16 | - | - |
| **T2DM-5** | 71.49 | 17.25 | 10.35 | 0.86 | 0.02 | 0.02 | - | - | - |
| **T2DM-6** | 62.40 | 26.72 | 10.43 | 0.37 | 0.05 | 0.02 | 0.02 | - | - |
| **MLE-1** | 68.54 | 26.85 | 4.14 | 0.35 | 0.04 | 0.08 | 0.01 | - | - |
| **MLE-2** | 52.15 | 16.14 | 30.95 | 0.67 | 0.03 | 0.01 | 0.04 | - | - |
| **MLE-3** | 74.37 | 19.61 | 5.66 | 0.27 | 0.03 | 0.02 | 0.05 | - | - |
| **MLE-4** | 73.44 | 20.06 | 6.07 | 0.35 | 0.02 | 0.06 | - | - | - |
| **MLE-5** | 79.58 | 16.99 | 3.08 | 0.27 | 0.02 | 0.04 | - | - | 0.02 |
| **MLE-6** | 76.58 | 16.21 | 6.86 | 0.28 | 0.03 | 0.02 | 0.01 | - | - |
