# Supplementary material for: Ethanol extract of mulberry leaves partially restores the composition of intestinal microbiota and strengthens liver glycogen fragility in type 2 diabetic rats
Source: BMC Complement Med Ther. 2021 Jun 14;21:172. doi: 10.1186/s12906-021-03342-x (PMC8204513; doi:10.1186/s12906-021-03342-x)
Supplement: Supplementary file 3 — Additional file 3. [file 12906_2021_3342_MOESM3_ESM.docx]

**Title**

Ethanol extract of mulberry leaves partially restores the composition of intestinal microbiota and strengthens liver glycogen fragility in type 2 diabetic rats

**Running Title**

Mulberry leaf extract and diabetes

Zhan-Zhong Liu^1,2,3,#^, Qing-Hua Liu^2,3,#^, Zhao Liu^4,#^, Jia-Wei Tang^5^, Eng-Guan Chua^6^, Fen Li^7^, Xuesong Xiong^7^, Meng-Meng Wang^2,3^, Peng-Bo Wen^5^, Xin-Yi Shi^8^, Xiang-Yu Xi^1^, Xiao Zhang^5,9,*^, Liang Wang^2,5,10,*^

**Supplementary Table 3** Composition of intestinal microbiota at genus level in each sample of the three groups, NC (n=6), T2DM (n=6), and MLE (n=6).

| **Genus** | **NC-1** | **NC-2** | **NC-3** | **NC-4** | **NC-5** | **NC-6** | **T2DM-1** | **T2DM-2** | **T2DM-3** | **T2DM-4** | **T2DM-5** | **T2DM-6** | **MLE-1** | **MLE-2** | **MLE-3** | **MLE-4** | **MLE-5** | **MLE-6** |
| --- | --- | --- | --- | --- | --- | --- | --- | --- | --- | --- | --- | --- | --- | --- | --- | --- | --- | --- |
| ***unclassified*** | 55.70 | 50.69 | 49.04 | 40.50 | 43.10 | 40.70 | 13.30 | 33.55 | 16.65 | 12.55 | 22.82 | 27.76 | 39.47 | 21.34 | 24.56 | 21.76 | 31.79 | 26.49 |
| ***Lactobacillus*** | 9.94 | 17.86 | 8.93 | 25.59 | 16.82 | 15.50 | 18.09 | 12.62 | 24.57 | 38.96 | 14.03 | 16.22 | 4.30 | 15.24 | 15.67 | 17.63 | 6.01 | 12.01 |
| ***Ruminococcus2*** | 0.06 | 0.01 | 0.11 | 0.08 | 0.04 | 0.12 | 8.57 | 11.19 | 29.56 | 6.14 | 36.61 | 25.78 | 31.14 | 12.66 | 37.44 | 36.27 | 31.52 | 36.11 |
| ***Bifidobacterium*** | 0.01 | 0.04 | - | - | 0.01 | - | 47.42 | 22.68 | 16.62 | 27.32 | 10.24 | 10.22 | 4.01 | 30.63 | 5.47 | 5.89 | 2.98 | 6.70 |
| ***Barnesiella*** | 8.45 | 10.27 | 10.40 | 8.63 | 10.61 | 12.16 | 1.61 | 3.05 | 2.35 | 2.99 | 2.30 | 2.49 | 6.05 | 2.65 | 3.87 | 3.70 | 1.82 | 3.72 |
| ***Bacteroides*** | 3.10 | 5.43 | 4.20 | 2.63 | 6.52 | 4.72 | 0.57 | 2.49 | 0.50 | 0.67 | 1.40 | 1.38 | 2.69 | 2.06 | 1.95 | 2.49 | 1.73 | 0.65 |
| ***Prevotella*** | 0.82 | 3.31 | 6.68 | 7.20 | 5.81 | 8.93 | 0.16 | 0.07 | 0.68 | 1.37 | 0.41 | 3.71 | 0.81 | 0.63 | 0.99 | 0.96 | 1.24 | 1.71 |
| ***Ruminococcus*** | 3.34 | 1.72 | 1.32 | 1.93 | 2.09 | 2.40 | 0.41 | 0.79 | 0.41 | 0.14 | 0.26 | 0.35 | 0.80 | 0.57 | 0.47 | 0.54 | 6.11 | 0.23 |
| ***Romboutsia*** | 1.58 | 0.78 | 1.21 | 1.52 | 1.50 | 1.24 | 1.39 | 1.39 | 2.09 | 3.90 | 1.21 | 2.07 | 1.10 | 0.94 | 0.11 | 0.15 | 1.84 | 0.28 |
| ***Clostridium IV*** | 1.41 | 0.95 | 1.05 | 0.39 | 0.97 | 0.61 | 0.76 | 2.09 | 0.47 | 0.75 | 1.46 | 0.58 | 0.75 | 0.87 | 1.15 | 0.40 | 1.93 | 1.08 |
| ***Clostridium XlVa*** | 2.14 | 0.45 | 2.26 | 0.81 | 0.40 | 1.90 | 0.16 | 0.36 | 0.37 | 0.12 | 1.25 | 1.27 | 0.50 | 0.48 | 0.48 | 0.57 | 2.25 | 1.60 |
| ***Intestinimonas*** | 2.45 | 1.43 | 2.29 | 1.08 | 1.01 | 1.64 | 0.26 | 0.85 | 0.19 | 0.22 | 0.16 | 0.46 | 0.51 | 0.71 | 0.51 | 0.69 | 0.25 | 0.57 |
| ***Turicibacter*** | 1.97 | 1.20 | 2.29 | 1.48 | 2.32 | 0.92 | 0.33 | 2.32 | 0.63 | 0.32 | 0.26 | 0.32 | 0.42 | 0.08 | 0.02 | 0.02 | 0.44 | 0.03 |
| ***Parasporobacterium*** | - | - | - | - | - | - | 2.20 | 1.64 | 0.86 | 0.41 | 1.66 | 2.03 | 0.57 | 2.63 | 0.83 | 0.91 | 1.09 | 0.54 |
| ***Allobaculum*** | 0.07 | 0.05 | 0.04 | 0.07 | 0.13 | 0.10 | 1.43 | 0.22 | 1.18 | 0.77 | 0.11 | 0.32 | 0.24 | 2.78 | 2.40 | 2.63 | 0.16 | 0.61 |
| ***Blautia*** | 0.05 | 0.10 | 0.16 | 0.16 | 0.12 | 0.09 | 0.37 | 0.64 | 0.37 | 0.29 | 0.86 | 1.26 | 1.40 | 0.65 | 0.29 | 0.50 | 4.33 | 0.16 |
| ***Roseburia*** | 0.13 | 0.02 | 0.25 | 0.20 | 0.17 | 0.57 | 0.24 | 0.07 | 0.16 | 0.05 | 0.56 | 0.20 | 0.82 | 1.52 | 0.49 | 0.31 | 0.61 | 3.13 |
| ***Weissella*** | - | - | - | - | - | - | 0.55 | 0.62 | 0.26 | 0.97 | 0.81 | 0.33 | 1.77 | 0.76 | 0.73 | 1.09 | 0.09 | 0.06 |
| ***Alistipes*** | 1.07 | 0.55 | 0.95 | 0.85 | 0.49 | 0.60 | 0.02 | 0.14 | 0.07 | 0.05 | 0.04 | 0.11 | 0.04 | 0.06 | 0.06 | 0.17 | 0.02 | 0.06 |
| ***Parasutterella*** | 0.19 | 0.71 | 0.63 | 0.72 | 1.16 | 0.96 | 0.11 | 0.06 | 0.20 | 0.10 | 0.12 | 0.25 | 0.09 | 0.49 | 0.11 | 0.16 | 0.04 | 0.02 |
| ***Clostridium sensu stricto*** | 0.61 | 0.12 | 0.20 | 0.50 | 0.31 | 0.30 | 0.35 | 0.91 | 0.47 | 0.11 | 0.08 | 0.10 | 0.24 | 0.08 | 0.05 | 0.11 | 0.22 | 0.11 |
| ***Parabacteroides*** | 0.29 | 0.63 | 0.83 | 0.41 | 0.64 | 0.47 | 0.08 | 0.43 | 0.06 | 0.04 | 0.20 | 0.12 | 0.28 | 0.09 | 0.14 | 0.38 | 0.17 | 0.05 |
| ***Alloprevotella*** | 0.09 | 0.13 | 1.28 | 1.34 | 1.19 | 0.59 | 0.01 | 0.01 | - | 0.30 | - | 0.01 | - | 0.01 | 0.03 | 0.17 | - | 0.03 |
| ***Lachnospiracea_incertae_sedis*** | 0.51 | 0.20 | 0.23 | 0.51 | 0.55 | 0.36 | 0.06 | 0.01 | 0.06 | 0.02 | 0.10 | 0.22 | 0.05 | 0.06 | 0.11 | 0.05 | 0.22 | 0.34 |
| ***Oscillibacter*** | 0.24 | 0.14 | 0.59 | 0.35 | 0.14 | 0.74 | 0.03 | 0.03 | 0.03 | 0.03 | 0.06 | 0.11 | 0.15 | 0.04 | 0.09 | 0.03 | 0.05 | 0.62 |
| ***Anaerovorax*** | 0.50 | 0.28 | 0.66 | 0.22 | 0.31 | 0.26 | 0.06 | 0.12 | 0.04 | 0.02 | 0.06 | 0.08 | 0.07 | 0.09 | 0.06 | 0.04 | 0.11 | 0.13 |
| ***Phascolarctobacterium*** | - | - | - | - | - | - | 0.11 | 0.15 | 0.01 | 0.01 | 0.47 | 1.00 | 0.12 | 0.51 | 0.49 | 0.18 | 0.64 | 0.42 |
| ***Flavonifractor*** | 0.38 | 0.21 | 0.28 | 0.18 | 0.16 | 0.22 | 0.08 | 0.04 | 0.15 | 0.02 | 0.04 | 0.09 | 0.11 | 0.06 | 0.27 | 0.05 | 0.04 | 0.68 |
| ***Helicobacter*** | 0.45 | 0.09 | 0.71 | 0.14 | 0.34 | 0.19 | 0.08 | 0.08 | 0.03 | 0.04 | 0.65 | 0.07 | 0.04 | 0.01 | 0.06 | 0.04 | 0.13 | 0.07 |
| ***Sporobacter*** | 0.13 | 0.28 | 0.24 | 0.08 | 0.11 | 0.04 | 0.05 | 0.15 | 0.03 | 0.01 | 0.08 | 0.08 | 0.10 | 0.06 | 0.11 | 1.13 | 0.13 | 0.06 |
| ***Pseudoflavonifractor*** | 0.29 | 0.17 | 0.39 | 0.10 | 0.23 | 0.24 | 0.07 | 0.06 | 0.11 | 0.05 | 0.03 | 0.05 | 0.16 | 0.03 | 0.01 | 0.03 | 0.10 | 0.23 |
| ***Clostridium III*** | 0.44 | 0.51 | 0.22 | 0.27 | 0.23 | 0.15 | - | 0.07 | - | 0.02 | - | - | - | - | - | - | - | 0.01 |
| ***Eubacterium*** | 0.27 | 0.21 | 0.35 | 0.28 | 0.18 | 0.30 | 0.01 | 0.03 | 0.01 | 0.03 | 0.07 | 0.04 | 0.03 | 0.03 | 0.05 | 0.03 | 0.06 | 0.06 |
| ***Dorea*** | 0.07 | 0.03 | 0.43 | 0.15 | 0.69 | 0.88 | - | 0.01 | - | - | - | 0.02 | - | 0.02 | 0.01 | - | - | 0.01 |
| ***Faecalibacterium*** | 0.52 | 0.06 | 0.24 | 0.13 | 0.14 | 0.23 | 0.02 | 0.03 | 0.04 | 0.02 | 0.04 | 0.03 | 0.02 | 0.03 | 0.02 | 0.03 | 0.02 | 0.08 |
| ***Saccharibacteria_genera_incertae_sedis*** | 0.37 | 0.28 | 0.20 | 0.10 | 0.21 | 0.10 | 0.04 | 0.10 | 0.03 | 0.12 | 0.02 | 0.05 | 0.04 | 0.03 | 0.03 | 0.02 | 0.02 | 0.03 |
| ***Coprococcus*** | 0.10 | 0.01 | 0.07 | 0.07 | 0.04 | 0.11 | 0.11 | 0.09 | 0.10 | 0.06 | 0.78 | 0.11 | 0.05 | 0.17 | 0.06 | 0.08 | 0.27 | 0.09 |
| ***Paraprevotella*** | 0.08 | 0.11 | 0.25 | 0.37 | 0.32 | 0.42 | - | 0.01 | 0.01 | 0.01 | 0.01 | - | 0.02 | 0.01 | 0.02 | 0.03 | 0.01 | 0.02 |
| ***Lactococcus*** | 0.01 | - | 0.02 | 0.02 | 0.01 | 0.01 | 0.10 | 0.07 | 0.09 | 0.23 | 0.03 | 0.02 | 0.10 | 0.02 | 0.05 | 0.05 | 0.69 | 0.32 |
| ***Enterococcus*** | 0.01 | - | - | - | - | - | 0.13 | 0.11 | 0.07 | 0.22 | 0.04 | 0.02 | 0.27 | 0.20 | 0.06 | 0.08 | 0.04 | - |
| ***Escherichia/Shigella*** | 0.20 | 0.04 | 0.03 | - | 0.24 | 0.08 | 0.01 | 0.05 | 0.02 | 0.07 | 0.07 | 0.03 | 0.05 | 0.05 | 0.01 | 0.01 | 0.06 | 0.01 |
| ***Clostridium XlVb*** | 0.03 | 0.08 | 0.15 | 0.11 | 0.16 | 0.14 | 0.03 | 0.01 | 0.01 | 0.01 | 0.01 | 0.01 | 0.01 | 0.02 | 0.02 | 0.02 | 0.02 | 0.10 |
| ***Pseudomonas*** | 0.27 | 0.07 | - | - | - | - | 0.03 | 0.05 | - | - | - | - | 0.02 | 0.03 | - | - | - | - |
| ***Olsenella*** | 0.01 | 0.04 | 0.01 | 0.01 | 0.01 | 0.04 | 0.09 | 0.01 | 0.06 | 0.04 | - | 0.03 | 0.02 | 0.21 | 0.04 | 0.04 | - | 0.08 |
| ***Asaccharobacter*** | 0.04 | 0.04 | 0.04 | 0.10 | 0.06 | 0.08 | 0.05 | 0.02 | 0.02 | 0.05 | 0.05 | 0.05 | 0.04 | 0.05 | 0.03 | 0.06 | 0.02 | 0.04 |
| ***Peptococcus*** | 0.01 | 0.04 | 0.07 | 0.03 | 0.02 | 0.14 | 0.07 | 0.06 | 0.05 | 0.04 | 0.07 | 0.12 | 0.02 | 0.01 | 0.01 | 0.02 | 0.04 | 0.01 |
| ***Macellibacteroides*** | 0.01 | - | 0.04 | 0.03 | 0.01 | 0.04 | 0.04 | 0.04 | 0.04 | 0.01 | 0.06 | 0.06 | 0.08 | 0.04 | 0.09 | 0.09 | 0.02 | 0.09 |
| ***Acetatifactor*** | 0.03 | 0.02 | 0.08 | 0.07 | 0.02 | 0.09 | 0.01 | - | - | 0.01 | 0.03 | 0.03 | 0.04 | 0.03 | 0.04 | 0.03 | 0.01 | 0.13 |
| ***Gemmiger*** | 0.18 | 0.02 | 0.09 | 0.03 | 0.02 | 0.03 | 0.01 | - | 0.01 | - | - | - | 0.01 | 0.01 | 0.01 | 0.01 | - | 0.02 |
| ***Coprobacter*** | 0.03 | - | 0.05 | 0.09 | 0.04 | 0.04 | 0.03 | 0.06 | 0.02 | 0.01 | 0.05 | 0.03 | 0.07 | - | 0.03 | 0.01 | 0.04 | 0.03 |
| ***Anaerotruncus*** | 0.10 | 0.01 | 0.02 | 0.03 | 0.02 | 0.03 | 0.02 | 0.01 | 0.01 | 0.01 | 0.03 | 0.01 | 0.05 | 0.02 | 0.04 | 0.01 | 0.02 | 0.05 |
| ***Odoribacter*** | 0.11 | 0.04 | 0.05 | 0.05 | 0.03 | 0.06 | - | 0.01 | - | - | - | - | - | - | 0.01 | 0.02 | 0.01 | 0.01 |
| ***Desulfovibrio*** | 0.06 | 0.01 | 0.02 | 0.01 | - | 0.01 | 0.10 | 0.01 | 0.08 | - | - | - | - | 0.01 | 0.01 | 0.01 | - | 0.01 |
| ***Papillibacter*** | 0.15 | - | 0.02 | - | 0.01 | 0.03 | 0.01 | - | - | - | - | - | 0.03 | - | - | - | - | - |
| ***Streptococcus*** | 0.04 | 0.03 | 0.04 | 0.02 | 0.03 | 0.02 | 0.01 | 0.01 | 0.02 | 0.01 | 0.01 | 0.03 | 0.03 | 0.02 | - | 0.01 | 0.04 | 0.01 |
| ***Erysipelotrichaceae_incertae_sedis*** | 0.08 | 0.03 | 0.02 | 0.02 | 0.03 | 0.05 | - | - | - | - | 0.01 | - | - | - | 0.01 | 0.01 | 0.01 | 0.02 |
| ***Defluviitalea*** | - | 0.04 | 0.01 | 0.01 | 0.01 | 0.01 | 0.02 | 0.04 | - | - | 0.04 | 0.04 | 0.02 | 0.02 | - | 0.01 | 0.05 | 0.01 |
| ***Butyricimonas*** | 0.01 | 0.06 | 0.05 | 0.02 | 0.03 | 0.01 | 0.01 | 0.01 | - | - | 0.01 | 0.02 | 0.01 | 0.01 | 0.02 | 0.02 | - | - |
| ***Akkermansia*** | 0.03 | - | 0.01 | - | - | - | 0.01 | 0.01 | - | 0.16 | - | 0.02 | 0.01 | 0.04 | 0.05 | - | - | 0.01 |
| ***Anaerofilum*** | 0.06 | 0.01 | 0.02 | 0.02 | 0.01 | 0.02 | 0.01 | 0.01 | - | 0.01 | 0.01 | 0.01 | 0.01 | 0.01 | 0.02 | - | 0.02 | 0.02 |
| ***Sphingomonas*** | 0.08 | 0.03 | - | - | - | - | 0.01 | 0.02 | - | - | - | - | 0.01 | 0.01 | - | - | - | - |
| ***Christensenella*** | 0.01 | 0.01 | 0.01 | 0.03 | 0.01 | 0.02 | 0.01 | 0.04 | - | - | 0.02 | 0.02 | 0.01 | - | 0.02 | 0.02 | 0.03 | 0.01 |
| ***Clostridium XVIII*** | 0.01 | 0.04 | 0.03 | 0.01 | 0.02 | 0.03 | - | - | - | - | 0.02 | 0.02 | - | - | - | - | 0.09 | - |
| ***Hafnia*** | 0.10 | - | - | - | - | - | - | 0.03 | - | - | - | - | - | - | - | - | - | - |
| ***Staphylococcus*** | - | - | - | - | 0.01 | 0.01 | - | - | - | - | - | - | 0.01 | 0.02 | 0.05 | 0.11 | - | 0.01 |
| ***Marvinbryantia*** | 0.02 | - | 0.02 | 0.05 | - | 0.05 | - | - | - | - | - | - | - | - | - | - | - | 0.02 |
| ***Collinsella*** | 0.04 | 0.07 | - | - | - | - | - | - | - | - | - | - | - | - | - | - | - | - |
| ***Anaerostipes*** | - | - | - | - | - | 0.08 | 0.01 | 0.01 | 0.01 | - | - | - | - | 0.01 | 0.01 | 0.01 | - | 0.05 |
| ***Vampirovibrio*** | - | 0.04 | 0.02 | 0.05 | 0.01 | 0.03 | - | - | - | - | - | - | - | - | - | 0.03 | 0.01 | - |
| ***Acetanaerobacterium*** | 0.03 | 0.01 | 0.01 | - | 0.01 | 0.02 | - | - | - | - | 0.01 | 0.02 | 0.01 | 0.01 | - | - | - | 0.02 |
| ***Citrobacter*** | 0.07 | - | - | - | - | - | - | 0.01 | - | - | - | - | - | - | - | - | - | 0.01 |
| ***Mogibacterium*** | - | - | - | - | - | - | - | 0.05 | - | - | 0.02 | - | - | - | - | - | 0.11 | - |
| ***Corynebacterium*** | - | - | - | - | - | - | - | - | 0.01 | 0.01 | - | 0.01 | 0.02 | 0.02 | 0.05 | 0.04 | 0.01 | - |
| ***Murimonas*** | - | - | - | - | 0.01 | 0.01 | - | 0.01 | - | - | 0.05 | 0.05 | - | - | - | 0.01 | - | 0.01 |
| ***Holdemania*** | 0.03 | 0.01 | 0.02 | 0.01 | 0.01 | 0.01 | - | - | - | - | 0.01 | 0.01 | - | - | - | - | - | - |
| ***Tannerella*** | 0.04 | - | - | - | 0.01 | - | - | - | - | - | - | 0.01 | - | - | 0.01 | 0.01 | - | - |
| ***Raoultella*** | 0.04 | - | - | - | - | - | - | - | - | - | - | - | 0.01 | - | - | 0.01 | - | - |
| ***Butyricicoccus*** | - | - | 0.01 | - | 0.01 | - | - | 0.01 | - | - | - | 0.01 | 0.01 | - | 0.01 | - | 0.01 | 0.01 |
| ***Rothia*** | 0.01 | 0.02 | - | - | 0.01 | 0.01 | - | - | - | - | - | 0.01 | - | - | - | - | 0.02 | - |
| ***Stomatobaculum*** | 0.03 | - | 0.01 | - | - | 0.01 | - | - | - | - | - | - | - | - | - | - | - | - |
| ***Enterorhabdus*** | 0.02 | 0.01 | - | 0.01 | 0.01 | - | 0.01 | - | - | - | - | 0.01 | - | - | - | - | - | - |
| ***Coprobacillus*** | - | 0.02 | - | 0.01 | 0.01 | 0.02 | - | - | - | - | - | - | - | - | - | - | 0.04 | - |
| ***Hydrogenoanaerobacterium*** | 0.01 | 0.01 | 0.01 | 0.01 | - | - | - | - | 0.01 | - | - | - | 0.01 | - | 0.01 | - | - | 0.01 |
| ***Lachnospira*** | - | - | - | - | - | - | - | - | 0.03 | - | 0.01 | 0.01 | - | - | - | - | 0.04 | 0.01 |
| ***Paraeggerthella*** | 0.01 | - | 0.01 | 0.01 | 0.01 | 0.01 | 0.01 | - | - | - | - | - | - | - | - | - | - | - |
| ***Anaerofustis*** | - | - | - | - | 0.01 | - | - | - | - | - | - | - | 0.01 | 0.01 | 0.01 | 0.01 | 0.02 | 0.01 |
| ***Aestuariispira*** | 0.01 | - | - | - | - | - | - | 0.03 | - | - | - | - | 0.01 | - | - | - | - | - |
| ***Falsiporphyromonas*** | 0.02 | - | 0.02 | 0.01 | - | - | - | - | - | 0.01 | - | - | - | - | - | - | - | - |
| ***Anaeroplasma*** | 0.01 | 0.01 | - | 0.02 | 0.02 | 0.01 | - | - | - | - | - | - | - | - | - | - | - | - |
| ***Veillonella*** | - | 0.02 | - | - | 0.01 | - | - | - | - | - | - | - | - | - | - | - | 0.01 | - |
| ***Aerococcus*** | - | - | - | - | - | - | - | - | - | 0.03 | 0.01 | - | - | 0.01 | - | - | - | - |
| ***Coriobacterium*** | 0.01 | - | - | - | - | - | - | - | - | - | - | - | - | 0.01 | - | - | - | - |
| ***Jeotgalicoccus*** | 0.01 | 0.01 | - | - | 0.01 | - | - | - | - | - | - | - | - | - | - | - | - | - |
| ***Anaerobacterium*** | 0.01 | 0.02 | - | - | - | - | - | - | - | - | - | - | - | - | - | - | - | - |
| ***Catabacter*** | - | - | - | - | - | - | - | 0.01 | - | - | - | - | - | - | - | - | 0.01 | - |
| ***Acetivibrio*** | - | - | 0.01 | - | - | - | - | - | - | - | - | - | - | - | 0.01 | - | - | - |
| ***Gracilibacter*** | - | - | - | - | - | - | - | - | - | - | - | - | - | - | 0.01 | - | - | - |
| ***Kurthia*** | - | - | - | - | - | - | 0.01 | - | - | - | - | - | - | - | - | - | - | - |
| ***Acetitomaculum*** | - | - | - | - | - | - | - | - | - | - | - | 0.01 | - | - | 0.01 | - | - | - |
| ***Asticcacaulis*** | 0.01 | - | - | - | - | - | - | - | - | - | - | - | - | - | - | - | - | - |
| ***Parvibacter*** | - | - | - | - | - | - | - | - | - | - | - | 0.01 | - | - | - | - | - | - |
| ***Actinomyces*** | - | - | - | - | - | - | - | - | 0.01 | - | - | - | - | - | - | - | - | - |
| ***Fusobacterium*** | - | - | - | - | - | - | - | - | - | - | - | - | - | - | - | - | 0.02 | - |
| ***Polynucleobacter*** | - | - | - | - | - | - | - | - | - | - | - | - | - | - | - | - | - | - |
| ***Haliscomenobacter*** | - | - | - | - | - | - | - | - | - | - | - | - | - | - | - | - | - | - |
| ***Acinetobacter*** | - | - | - | - | - | - | - | - | - | - | - | - | - | - | - | - | - | - |
| ***Bilophila*** | - | - | - | - | - | - | - | - | - | - | - | - | - | - | - | - | - | - |
| ***Ferritrophicum*** | - | 0.01 | - | - | - | - | - | - | - | - | - | - | - | - | - | - | - | - |
